# A supervised machine learning tool to predict the bactericidal efficiency of nanostructured surface

**DOI:** 10.1186/s12951-024-02974-8

**Published:** 2024-12-03

**Authors:** Yaxi Chen, Hongyi Chen, Anthony Harker, Yuanchang Liu, Jie Huang

**Affiliations:** 1 Department of Mechanical Engineering, University College London, London, UK; 2 Department of Computer Science, University College London, London, UK; 3 Department of Physics & Astronomy, University College London, London, UK

**Keywords:** Machine learning, Nanotopography, Mechano-bactericidal activity, Antimicrobial properties

## Abstract

The emergence and rapid spread of multidrug-resistant bacterial strains is a growing concern of public health. Inspired by the natural bactericidal surfaces of lotus leaves and shark skin, increasing attention has been focused on the use of mechano-bactericidal methods to create surfaces with antibacterial and/or bactericidal effects. There have been several studies exploring the bactericidal effect of nanostructured surfaces under various combinations of parameters. However, the correlation and synergies between these factors still need to be clarified. Recently machine learning (ML), which enables prediction or decision-making based on data, has been used in the field of biomaterials with promising results. In this study, we explored ML in nanotechnology to investigate the antimicrobial potential of nanostructured surfaces. A dataset of nanostructured surfaces and their antimicrobial properties was built by extracting the published literature. Based on the literature review and the distribution of our dataset, 70% bactericidal efficiency was selected as a practical benchmark for our classification model that balances stringent bactericidal performance with achievable targets in diverse conditions. Subsequently, we developed an ML classification model, which demonstrated an 81% accuracy in its predictive capability. A regression model was further developed to predict the value of bactericidal efficiency for nanostructured surfaces. Feature importance analysis of the ML models suggested that nanotopographical features have a greater influence on bactericidal properties than material properties, thus providing insight into the principles of the mechano-bactericidal effect of nanostructured surfaces. Overall, this ML model tool could help researchers to effectively select and design the parameters of the surface structure prior to experimentation, thereby improving the timeliness and reducing the number of experiments and the associated costs.

**Graphical Abstract:**

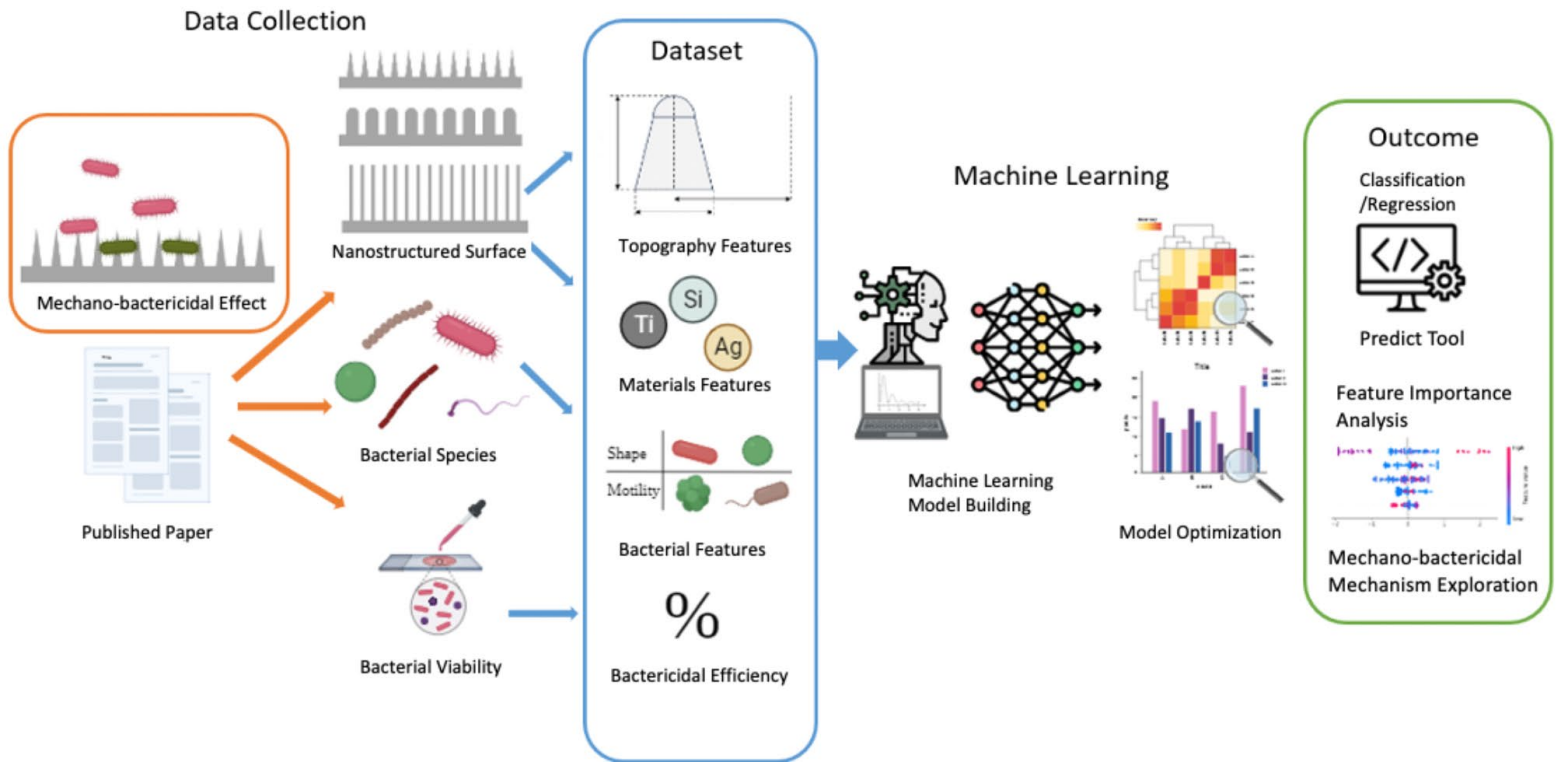

**Supplementary Information:**

The online version contains supplementary material available at 10.1186/s12951-024-02974-8.

## Introduction

In healthcare environments, infections can lead to increased morbidity, mortality, and healthcare costs, highlighting the need for stringent infection control practices [1]. Effective infection control measures are essential to prevent the spread of harmful pathogens and reduce the risk of nosocomial infections [2]. Within the context of bacterial contamination in medical and surgical fields, biofilm formation (surface-attached, structured microbial communities containing bacteria [3]) poses a significant challenge, as it can lead to the destruction of adjacent tissue, poor vascularization, implant loosening, detachment, and even dislocations [4]. These complications often arise during surgical procedures when implants become particularly vulnerable to bacterial contamination from the skin and mucous membranes. This susceptibility allows for the rapid progression of device-associated infections, as planktonic bacteria initially adhere to the implant interface and eventually evolve into biofilms, further exacerbating the problem. The use of coatings on implant surfaces containing antibiotics or other bactericidal agents, such as heavy metals like silver, copper, or zinc, has gained popularity as a method to prevent microbial colonization on implants [5]. However, the emergence of antibiotic-resistant bacteria and the limitations of conventional disinfection methods have led to a growing need for alternative solutions to address bacterial contamination effectively [6].

Over the past decade, researchers have gradually begun to focus on the use of mechano-physical methods to create surfaces with antibacterial and/or bactericidal effects. These mechano-bactericidal surfaces were bioinspired, and they were originally found on lotus leaves, shark skin, gecko skin, cicada wings, and dragonfly wings [7, 8]. The denticles (diamond-shaped scales covering the outer surface) and arrangement on shark skin create a superhydrophobic surface that exhibits good anti-fouling properties by creating a surface that is not conducive to bacterial adhesion. In contrast, the highly ordered array of nanopillars or nanocones of different sizes, heights, and spatial distribution on the wings of the cicada is capable of killing bacteria upon contact, creating a bactericidal effect [5, 9, 10]. It was proposed that the mechano-bactericidal activity of nanostructured surfaces is the result of the mechanical interaction between the bacteria and the nanopatterns, which is governed by surface geometry. This creates the possibility of drug-free bactericidal surfaces [11].

One of the generally accepted theories of the bactericidal mechanism of nanostructured surfaces is a biophysical interaction between the bacterial cell wall and the nanopatterns. The geometry (e.g., diameters, height) and spacing of nanopatterns influence how the cell wall is ruptured: Dense and blunt nanopillars tend to overstretch the membrane of adhered bacteria, whereas sharp ones tend to directly impale the cells [5]. In addition, similar nanostructured surfaces may have different bactericidal efficiencies for different bacteria because the membrane structures of Gram-negative and Gram-positive bacteria are considerably different: Gram-positive bacteria have an inner membrane and a thick peptidoglycan layer (20–100 nm), while Gram-negative bacteria have an outer and inner membrane and an intermediate peptidoglycan layer (only a few nanometres thick). Besides cell wall rupture, an alternative mode of Gram-negative cell wall disruption involves the separation of the cytoplasmic membrane from the cell wall, which may be mediated by adhesive extracellular polymeric substances (EPSs) and triggered by the forces originating from the movement of bacterial cells trapped on the crest of nanopillars [12]. There is now growing evidence that other factors may also be involved in the bactericidal mechanism of nanostructured surfaces. For example, it has been discovered that mechanical injury is not sufficient to kill the bacteria immediately due to the survival of the inner plasma membrane. Instead, such sublethal mechanical injury leads to apoptosis-like death in affected bacteria [10].

To date, these antibacterial and/or bactericidal nanostructured surfaces have been fabricated from a wide range of materials including silicon [13], metals (e.g., titanium [14], gold [15], and ZnO [16]), and polymers [17, 18]. The fabrication methods for these synthetic nanostructured surfaces often allow close control over the parameters that define the nanostructured surfaces (the height, spacing, and diameter of the nanopatterns) and lead to bactericidal efficiencies that frequently exceed those of natural nanostructured surfaces [19]. The key factors affecting mechano-bactericidal properties of the nanostructured surface can be divided into two aspects: [1] Topography: shape and size of nanopattern, surface roughness, and wettability [2, 5] Materials: elasticity, flexibility, hydrophobicity, and lipophilicity. The bactericidal efficiency of nanostructured surfaces varies depending on the specific type of bacteria they are targeting. Additionally, one crucial inquiry concerning the bactericidal properties of nanopatterns involves determining the ideal design parameters that can maximize their bactericidal effectiveness while minimizing any potential negative impacts such as cytotoxicity [20]. However, the factors that determine the precise bactericidal activity of nanostructured surfaces are intricate and remain largely unexplored, giving rise to numerous contentious conclusions. For example, wettability is an important parameter for the early attachment of bacteria to a surface, however, the exact relationship between hydrophobicity and bacterial adhesion remains debatable. It has been shown that E. coli adhesion levels are highest on moderately hydrophobic surfaces (WCA = 95°) and lowest on hydrophilic (WCA < 30°) and superhydrophobic (WCA > 120°) surfaces [21]. Whereas antifouling surfaces usually prevent the adhesion of bacteria through their superhydrophobic properties, the same is true for many nanostructured surfaces. Another example is the currently prevailing postulation suggesting that the extent of membrane stretch increases with increasing diameters of nanopillars, but that the effect is minimal when the diameter exceeds 20 nm [22]. However, there are also results from in silico studies showing that reducing the diameters from 30 to 10 nm can increase the strain on the bacterial envelope by 25% [23]. These show that individual parameters of nanostructured surface are often entangled with other parameters, which makes the principle of bactericidal activity ambiguous.

There have been several studies exploring the bactericidal effect of nanostructured surfaces under various combinations of parameters. Some researchers conducted a comprehensive analysis to examine the relationship between the bactericidal activity of bacterial species and the various parameters tested on nanostructured surfaces [24]. However, their analysis was limited to a single-factor examination, and although it revealed the impact of different factors on bactericidal performance, it did not elucidate the direct correlation and synergistic effects among these factors. Understanding the impact of these factors on bactericidal efficiency is of great significance because it can help researchers better design nanostructured surfaces with targeted bactericidal effects. Machine learning (ML) is a branch of artificial intelligence (AI) that involves the development of algorithms and models capable of learning from and making predictions or decisions based on data [25]. Employing data analysis techniques based on ML principles could be a useful solution to this problem. Therefore, ML models are increasingly being utilized to address a diverse range of challenges in biomedical science and material science research [26]. For example, ML models have been used for predicting the cytotoxicity of nanomaterials [27] and predicting nanoparticle delivery to tumours [28]. ML methods use mathematical and statistical tools to identify and utilize the connections within the data, allowing for the creation of intricate models to describe the system [29].

Although ML has demonstrated its potential for widespread application, only a few studies applied ML to the development of nanosurfaces. To the best of our knowledge, our study is the first to use ML techniques to predict the antimicrobial properties of nanostructured surfaces. We aim to use ML to aid the prediction of the bactericidal efficiency of nanostructured surfaces and determine the optimum parameters for nanostructured surfaces to achieve superior mechano-bactericidal properties. An essential advantage of utilizing ML is that it enables experimenters to precisely define the range of experimental parameters, thereby substantially reducing the time, resources, and laboratory animal usage involved in these endeavours. Furthermore, we focus on the feature importance of the ML model and interpretation, and explore the mechanisms of bactericidal activity on nanostructured surfaces.

## Results

### Data acquisition

After a comprehensive review of 2,919 publication literature, 45 papers were selected and considered relevant to this research. 293 different nanostructured surfaces were studied in terms of substrate material, nanostructure shape and size, and surface hydrophobicity. The raw dataset is provided in Table S5. Data distribution of experiment parameters in the database was visualized by histograms and kernel density estimation (KDE) plots (Fig. S1). As depicted in the figure, some outliers existed in the database. For example, most nanopatterns are found in the height range 0–6500 nm, but a few reached 32,000 nm.

**Fig. 1:**
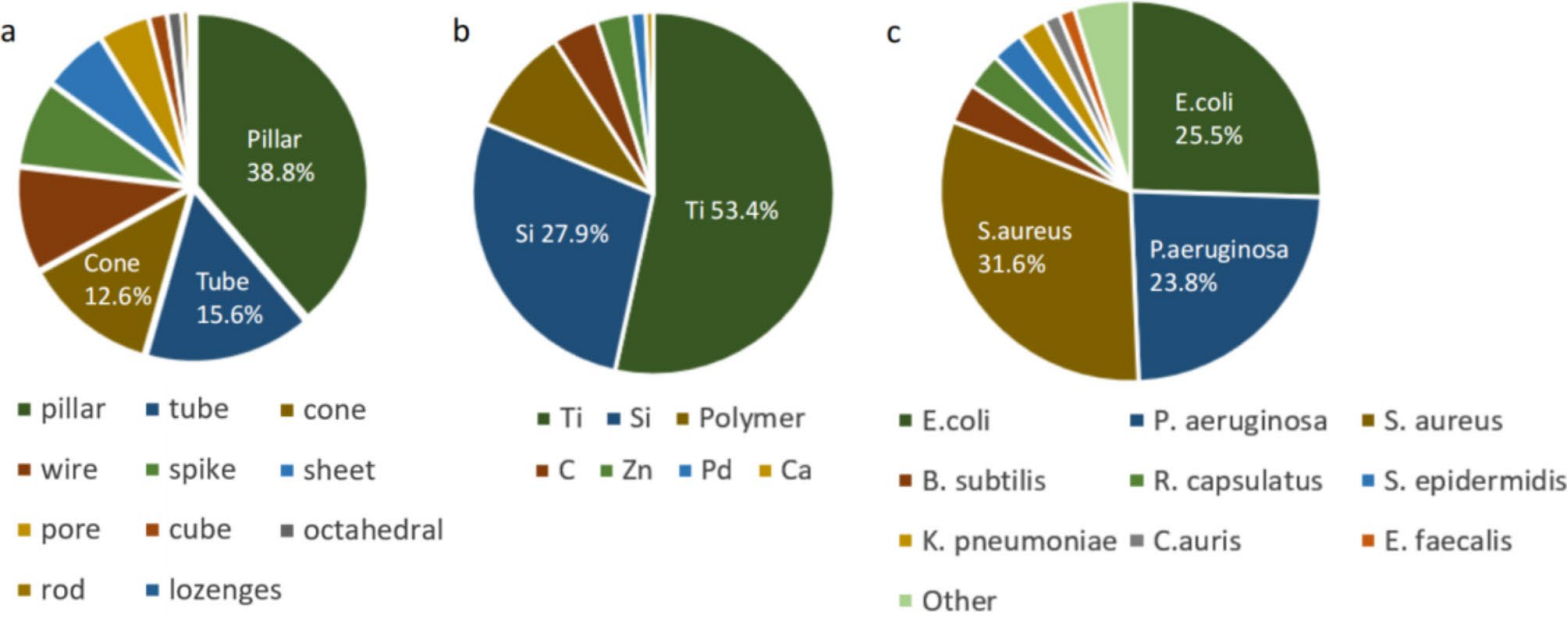
Data distributions of (**a**) Shape, (**b**) Materials, and (**c**) Bacteria Species

Titanium and silicon were the main choices of substrate materials for the fabrication of nanostructures. In contrast, the dataset is more evenly distributed among the bacterial species, centred on E. coli, P. aeruginosa, and S. aureus (Fig. 1). Of these, 121 were studies of Gram-positive bacteria and 173 were studies of Gram-negative bacteria. The nanopattern is also more evenly distributed in terms of shape, consisting mainly of pillar, but also partly of tube, cone, wire, spike, etc. There are 192 surfaces that are hydrophilic with a WCA ≤ 90° and 102 hydrophobic surfaces with a WCA > 90°. Details of the dataset can be found in the supplementary information.

### Data pre-processing

The primary dataset comprised 293 rows and 12 columns (11 inputs, 1 output). The input data consisted of diameter (nm), height (nm), spacing (nm), aspect ratio, surface roughness (nm), water contact angle (WCA) (°) reported in numeric values. Variables with nominal values included materials, shape of nanopatterns, bacteria Strain, Gram-stain type motility, and shape of bacteria as summarized in Tables 1, 2 and 3.

#### Input transformation

For materials of nanostructured surfaces, a simplified classification has been made due to the wide variety contained, e.g. Ti, Ti6Al4V, TiOH and TiO_2_ are classified as Ti-based.

**Table 1:** Summary of the primary and final input variables of materials parameters in data pre-processing

			Raw Data	Data Transformation	Dataset I
Category	Features	Type	Range or Labels		
Materials	Materials	Nominal	Ti, TiO_2_, Ti-OH, Ti6Al4V, Si, PET, CaP, ZnO, Pd, C, PS-b-P2VP, PS-block-PMMA	Simplified	Ti, Si, CaP, ZnO, Pd, C, Polymer

For nanotopogrpahy, the features such as diameter, height, spacing and aspect ratio are a good representation of the shape of the nanopattern, thus these features have been retained and the shape of the nanopattern has been eliminated. Surface roughness has approximately 90% or more missing values and was therefore excluded. Diameter, height, spacing, aspect ratio, and WCA all had less than 30% missing values and were retained for the next data imputation process.

**Table 2:** Summary of the primary and final input variables of nanotopography parameters in data pre-processing

			Raw Data	Data Transformation	Dataset I
Category	Features	Type	Range or Labels		
Nano topography	Shape	Nominal	Pillar, wire, pore, tube, cone, rod, octahedra, lozenges, squares, sheet, spike	Eliminated, use other features instead	-
	Diameter	Numeric	5.41–1000.00 (nm), NA	Selected	5.41–1000.00 (nm), NA
	Height		10.00-32000 (nm), NA		10.00-32000 (nm), NA
	Spacing		4.82–2000.00 (nm), NA		4.82–2000.00 (nm), NA
	Aspect Ratio		0.0003-4.00, NA		0.0003-4.00, NA
	Surface Roughness		0.69–2296.00 (nm), NA	Eliminated due to high NA	-
	Water Contact Angle		0-160 (°), NA	Selected	0-160 (°), NA

Similarly, the Gram-stain type, motility and shape are representative of the bacterial membrane structure, therefore these three features are selected as input and the name of bacterial species is eliminated.

**Table 3:** Summary of the primary and final input variables of bacteria features in data pre-processing

			Raw Data	Data Transformation	Dataset I
Category	Features	Type	Range or Labels		
Bacteria	Bacteria Strain	Nominal	E.coli, P. aeruginosa, S. aureus, S. epidermidis, B. subtilis, F. nucleatum, E. faecalis, P. gingivalis, L. monocytogenes, K. pneumoniae, C.auris, M. smegamatis	Eliminated, use other features instead	-
	Gram-stain type		Positive, Negative	Selected	Positive, Negative
	Motility		Motile, Nonmotile		Motile, Nonmotile
	Shape		Rod, Spherical		Rod, Spherical

#### Output transformation

We chose 70% as a threshold for our classification model building. This threshold is not arbitrarily set but is a reflection of a consensus within the nanobactericidal surface research community. We specifically referenced several articles that included nanobactericidal surfaces with more than five different parameters rather than a single morphology [30–38]. The distribution of bactericidal efficiency in these experiments was relatively uniform from 0 to 100%, with efficacious surfaces concentrated in the range of 60–80%, with 70% emerging as a practical benchmark that balances stringent bactericidal performance with achievable targets in diverse conditions. Thus, for regression models we kept the percentage of bactericidal efficiency as output features; for binary classification models we simplified the numeric bactericidal efficiency to 2 classes, i.e. whether it is a successful bactericidal surface.

### Classification model building

**Fig. 2:**
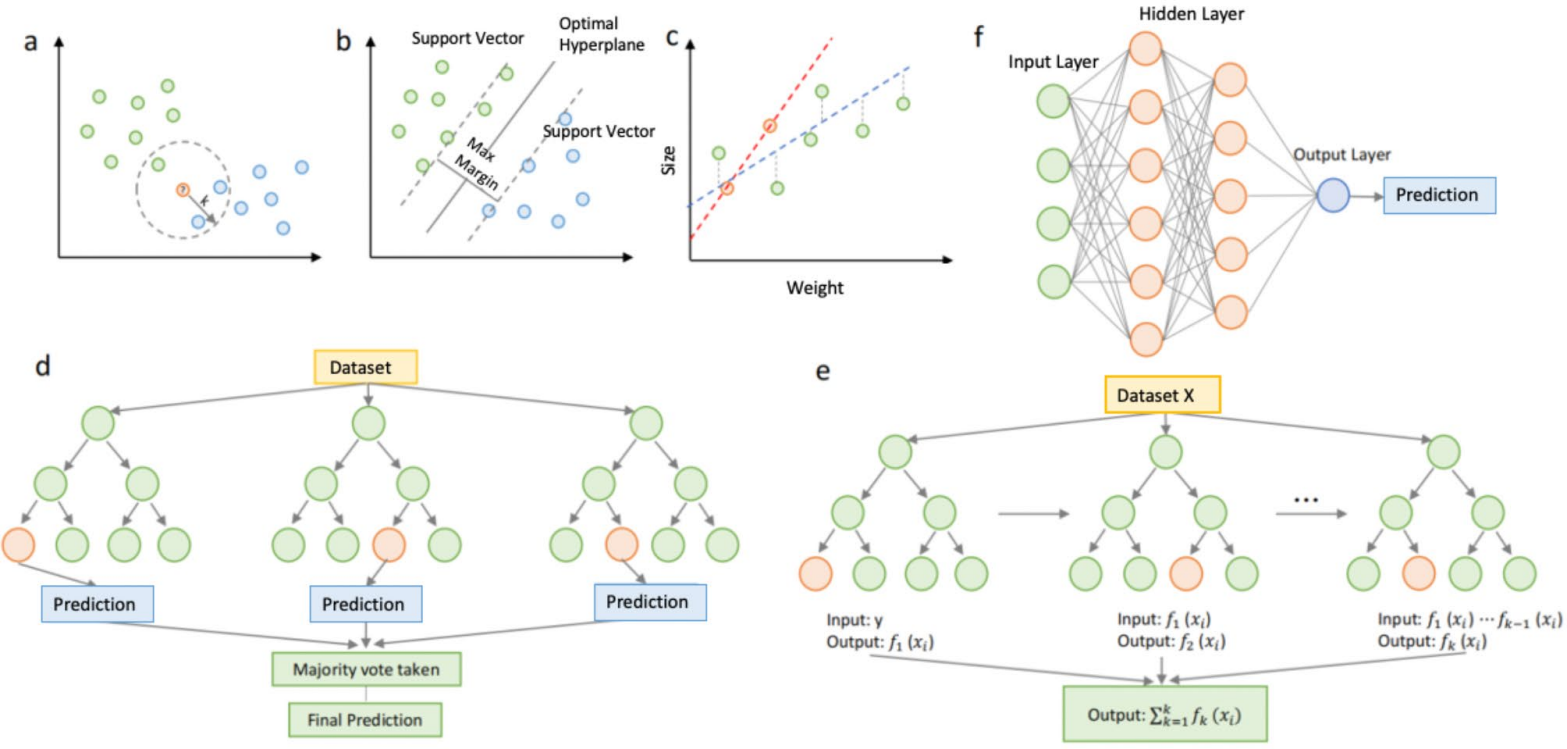
Illustration of the various ML methods used in the study. (**a**) K-nearest neighbour (KNN). (**b**) support vector machine (SVM). (**c**) ridge regression (RR). (**d**) Random Forest (RF) (**e**) Gradient boosting machine (GBM) and extreme gradient boosting (XGBoost). (**f**) Multilayer perceptron (MLP)

Model selection was critical for the accuracy of ML prediction, and we have chosen seven state-of-the-art algorithmic models for predicting the bactericidal efficiency, which included K-nearest neighbor (KNN), support vector machine (SVM), extreme gradient boost (XGBoost), gradient boosting machine (GBM), random forest (RF), multilayer perceptron (MLP) for classification modelling and ridge regression (RR), XGBoost, GBM, KNN for regression modelling [30–33]. A brief summary is illustrated in Fig. 2 and explained in Table 4.

**Table 4:** Enumeration dataset parameters for Ti-based nanostructured surfaces targeting Gram-negative bacteria

Features	Range	Step length
Height(nm)	(0,3600)	100
Diameter(nm)	(0,500)	50
Spacing(nm)	(0,800)	50
Aspect ratio	(0,4)	0.5
WCA(°)	(0,180)	10

#### Preliminary modelling

**Fig. 3:**
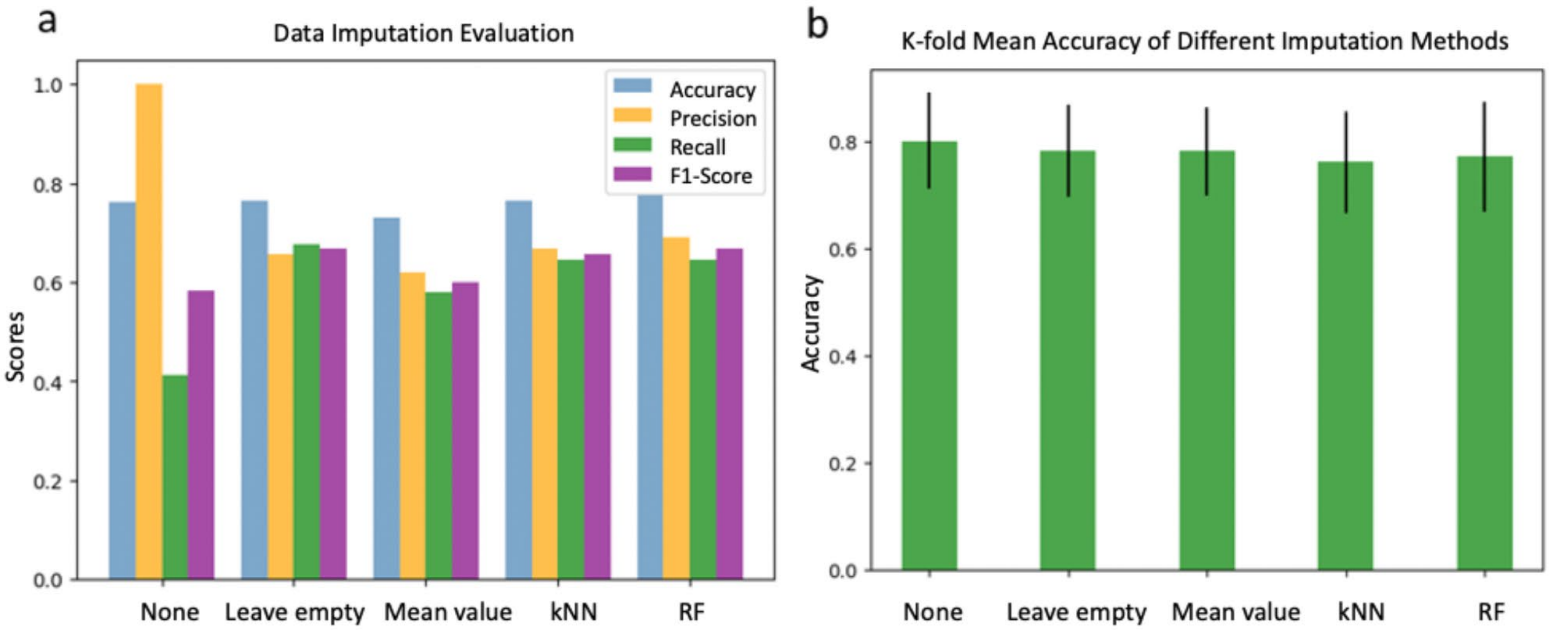
(**a**) Model performance of different data imputation methods evaluated by accuracy, precision, recall and F1 score, (**b**) Model performance of the different data imputation methods was assessed by the average accuracy obtained from 10-fold cross-validation. Error bars are from 10-fold cross-validation

After the initial screening, the missing values were imputed, using 5 different imputation strategies: None, Leave empty, Mean, KNN and RF (Explained in detail in the method section). Performances of different data imputation methods were compared, as shown in Fig. 3. It can be seen from the plots that different data imputation methods did affect model performance. Of the three active filling blank methods, RF performed the best, with the highest accuracy and F1 scores. The ‘None’ group had a high precision, which means the high credibility of a claim that a case is positive. However, it has a relatively low recall, which indicates some false positives. While the ‘leave empty’ group was more evenly split across all indicators. Further comparison of the results of their 10-fold cross-validation revealed that the mean accuracy of the different imputations showed little difference, stabilising at around 78%. Therefore, the ‘None’ group, the ‘leave empty’ group and the RF group were retained for the model building to further compare the impact of the data imputation methods on the performance of the models.

After data transformation the following three datasets were obtained for the model building step: Dataset I (*n* = 294, Leave empty group); Dataset II (*n* = 294, RF group); Dataset III (*n* = 140, None group). To further build a regression model to predict the bactericidal efficiency of successfully bactericidal surfaces, we extracted data for the RF group with a bactericidal efficiency greater than 70% as Dataset IV (*n* = 105).

#### Classification model building

**Fig. 4:**
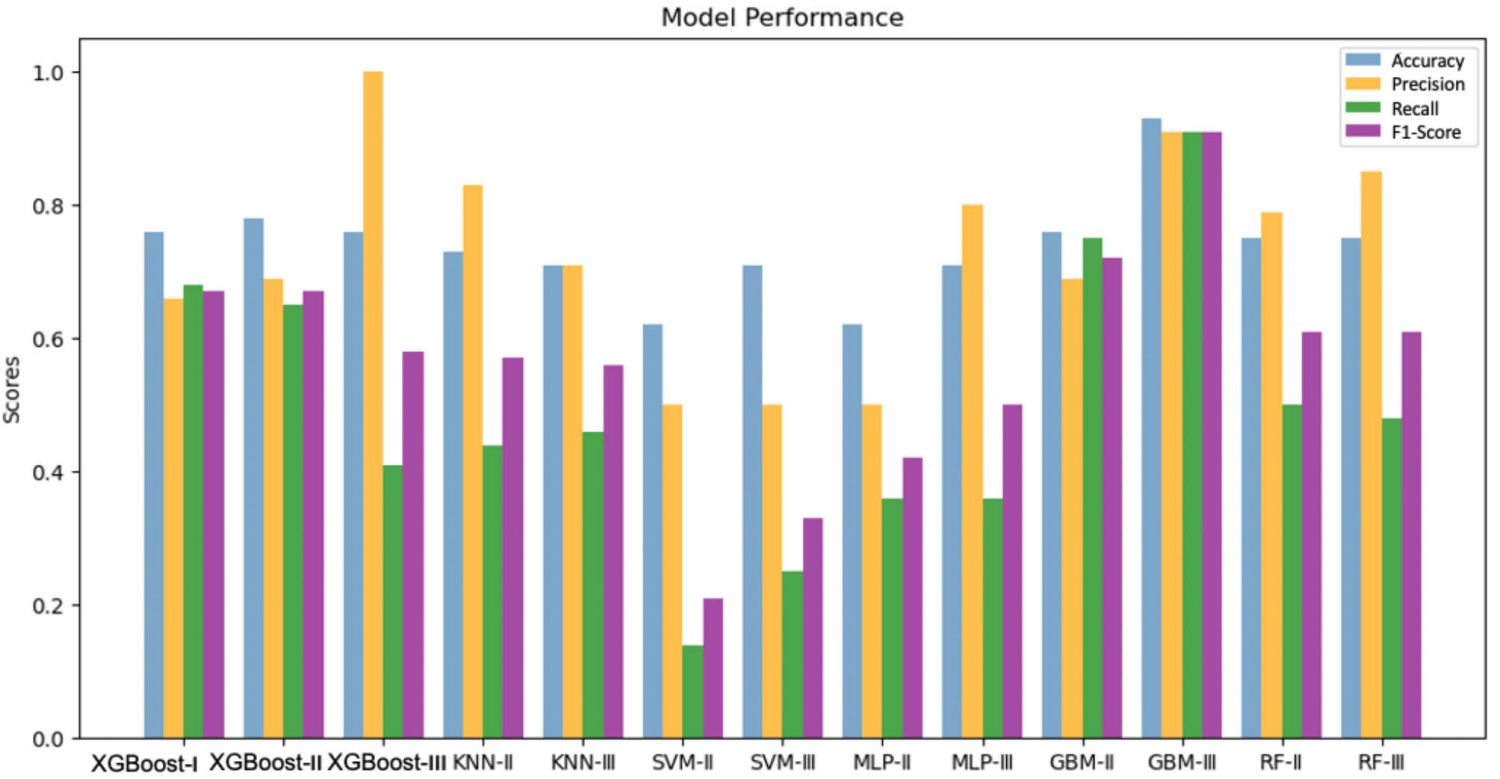
Classification model performance evaluated by accuracy, precision, recall and F1 score

Following preliminary modelling, we trained various classification models, and all model parameters were tuned to the best combination. By traversing all the model parameters, the best combination of parameters is selected (see Table S1). Model performance results are summarized in Fig. 4 and Table S3. The results suggest that the XGBoost and GBM models exhibit overall higher accuracy and less fluctuation, which indicated a more stable performance compared to the other algorithms employed (KNN, SVM, and MLP). It is quite interesting to note that most of the models built are high-accuracy but low-recall systems, returning very few results, but most of its predicted labels are correct when compared to the training labels. In comparison, XGBoost-I, II and GBM-III show high accuracy rates of 0.76, 0.78 and 0.93 respectively, and relatively high precision and recall.

We then compared the 10-fold validation results of the XGBoost and GBM models (Fig. S2). The GBM-III and XGBoost-III models have the highest average accuracy of 0.81 and 0.80 respectively, while XGBoost-III has smaller variation, representing greater precision. Therefore, the GBM-III model had the best overall performance, with an average accuracy of 0.81.

To further test the performance of the model with different data imputation methods, we compared the confusion matrixes to assess the performance of XGBoost models (XGBoost-I, II, III). The confusion matrices for XGBoost-I and II are identical (Fig. S3), indicating that using RF as a data imputation in this study is a non-inferior approach.

**Fig. 5:**
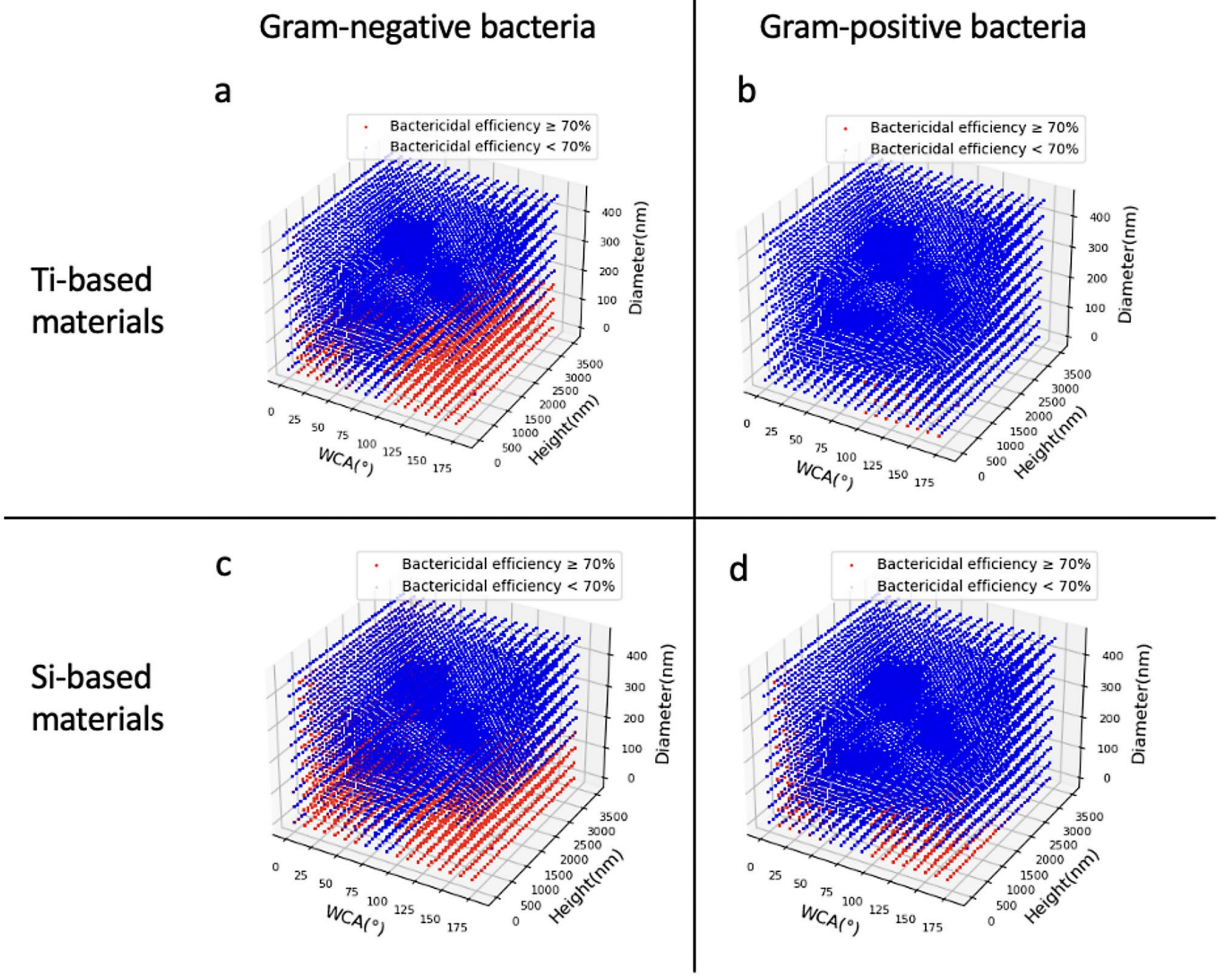
Bactericidal efficiency prediction map: (**a**) Ti-based nanostructured surfaces against Gram-negative bacteria, (**b**) Ti-based nanostructured surfaces against Gram-positive bacteria, (**c**) Si-based nanostructured surfaces against Gram-positive bacteria, and (**d**) Si-based nanostructured surfaces against Gram-negative bacteria

Subsequently, we utilised four new enumeration datasets (Ti-based nanostructured surfaces against Gram-negative bacteria, Ti-based nanostructured surfaces against Gram-positive bacteria, Si-based nanostructured surfaces against Gram-positive bacteria and Si-based nanostructured surfaces against Gram-negative bacteria with 829,448 datapoints in each dataset) to gain further insights into the nanostructured parameters and bactericidal efficiency of the nanostructure parameters and bactericidal efficiency. Based on the GBM-III models, we used the enumerated dataset to create a bactericidal efficiency map (Fig. 5). According to the figure, most of the high bactericidal efficiency surfaces, both Ti-based and Si-based materials, have polar WCAs, i.e., superhydrophilic and superhydrophobic. The nanostructured surfaces are overall more efficient in bactericidal activities for Gram-negative bacteria than for Gram-positive bacteria. In addition, the diameter of highly bactericidal surfaces is typically less than 200 nm.

### Feature importance analysis and model interpretation

#### Overview of feature importance

**Fig. 6:**
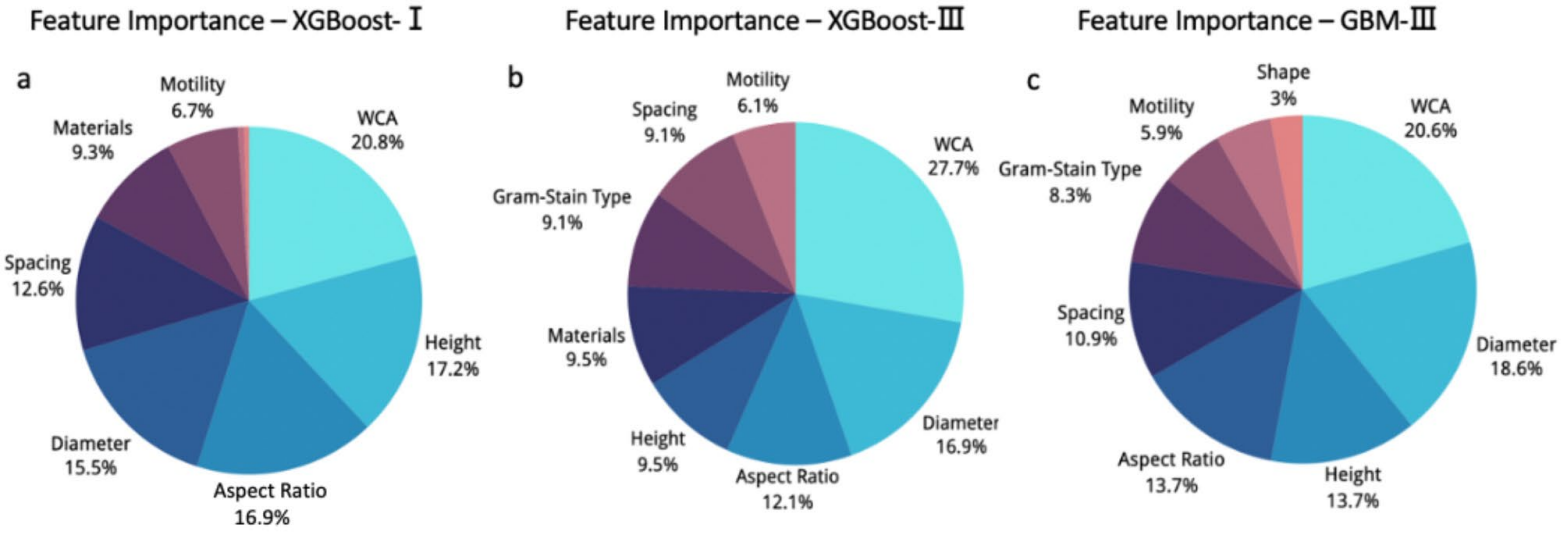
Feature importance distribution of (**a**) XGBoost-I, (**b**) XGBoost-III, (**c**) GBM-III model

Interpreting the model provides valuable insights into its learning characteristics. Feature importance learnt by the GBM-III model was plotted to represent the ML’s interpretation of the correlation between different features and bactericidal efficiency. The feature importance of the XGBoost-I, III; models were also analysed and used to compare the differences between the conclusions drawn under the different algorithms. The feature importance analysis for both models yielded similar conclusions (Fig. 6), showing that the top four importance rankings for both models were WCA, height, diameter and aspect ratio, all of which are features of nanotopography. This suggests that nanotopography is indeed the main factor dominating the bactericidal activity of nanostructured surfaces, which is also consistent with the mechano-bactericidal concept mentioned previously. For WCA, the feature importance is 20.8%, 27.7%, and 20.6% in the XGBoost-I, III; and GBM-III models, respectively. Although the majority of surfaces in the dataset were hydrophilic, the least-tested hydrophobic surfaces have shown higher success rates than their hydrophilic counterparts. The possible reason is that hydrophobic and hydrophilic surfaces have different mechanisms of bacterial inhibition, as mentioned previously, one preventing bacteria from adhering and the other killing them when they do, but the different inhibition mechanisms achieve the same purpose.

#### Model interpretation for topographical features

**Fig. 7:**
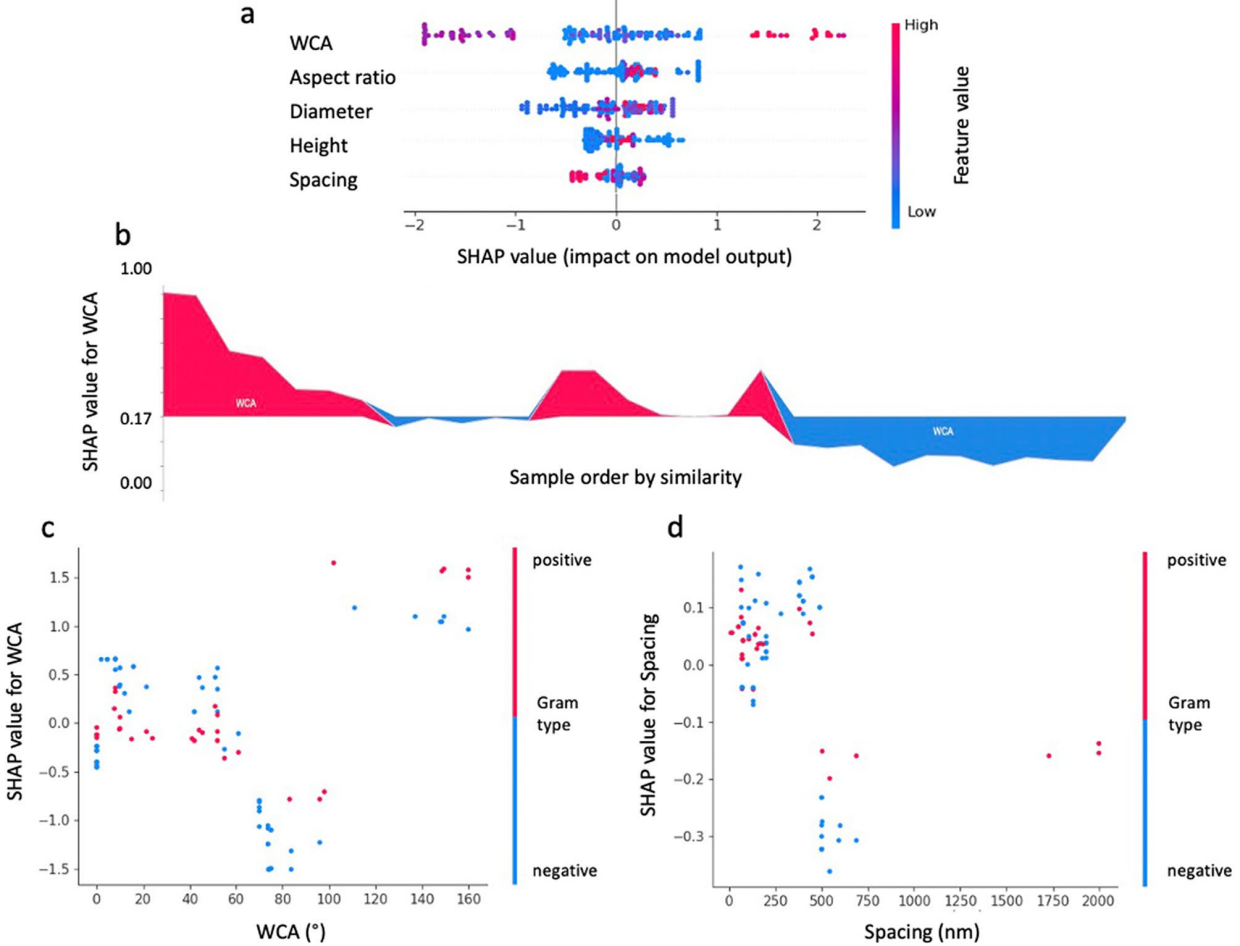
SHAP values analysis summary for XGBoost-III model. (**a**) SHAP values of different features show their contributions to the model output on the local scale. Impact: The horizontal location shows whether the effect of that value is associated with a higher or lower prediction; Original value: Colour shows whether that variable is high (in red) or low (in blue) for that observation; (**b**) SHAP summary force plot for WCA effects; SHAP dependence plots articulate the intricate relationship between the (**c**) WCA and Gram types, and (**d**) Spacing and Gram types

Figure 7 shows the Shapley additive explanations (SHAP) of topographical features. SHAP values is a unified framework to interpret ML predictions proposed by Lundberg and Lee [30], to describe how much each feature contributes to the predictions. In this ML model, the SHAP and feature values of the WCA are evenly distributed on the x-axis (Fig. 7a), while it can be concluded from the distribution of high feature value points that high WCA has a certain positive effect on bactericidal efficiency. Figure 7b elaborates on the variability in the impact of WCA on the model’s output across different samples. The analysis highlights that WCA values contributing positively to the model’s output predominantly fall within the ranges of 0–10 degrees or 160–180 degrees, as indicated by the red zones in the plot. These ranges correspond to surfaces that are extremely hydrophilic or hydrophobic, respectively, both of which are considered beneficial for bactericidal activity. Conversely, WCA values situated around the median, predominantly encapsulated within the blue zones of the plot, are associated with a negative impact on the output value. This suggests that surfaces with median WCA values may represent a less effective or undesirable range for bactericidal applications, indicating a complex relationship between surface wettability and bactericidal efficiency that is dependent on the extremity of the hydrophilic or hydrophobic nature of the surface.

Height and diameter are directly related to the bacteria-nanopattern contact area, while the tip size of the nanopattern is very important as it is the first point of contact between the bacteria and the surface [43]. The ML model shows that both diameter and height are positively correlated with bactericidal efficiency. Some studies based on analytical models support our conclusions, which suggest that a larger radius provides a wider contact area, driving the suspended region of the membrane to attempt to accommodate the change in the perimeter by stretching and eventually rupturing [23, 44]. However, smaller tip radius could induces higher pressure on the bacterial membrane, enhancing the bactericidal effect of the nanostructured surface [5].

The SHAP values for aspect ratio indicate that high aspect ratios have a positive effect on bactericidal efficiency. This is in line with Linklater et al. study [22], which demonstrated that the flexibility of a high aspect ratio structure enhances the elastic energy storage of the nanostructure and releases this energy through bending when in contact with bacteria, thereby increasing the bactericidal activity of the nanostructured surface.

#### Model interpretation for material properties and bacterial species

It is noteworthy that the material properties of the nanostructured surface account for a small proportion of the feature importance. This corresponds to the mechanisms revealed from some experimental approaches, i.e. the mechano-bactericidal mechanism on nanostructured surfaces is independent of chemical effects, as the functionality (bactericidal ability) was shown to persist across materials [7]. However, recent studies have suggested that biological and chemical processes also play a synergistic role in the bactericidal activity of nanostructured surfaces [45–47]. For example, Jenkins et al. proposed a synergistic ROS-mediated mechanism of mechano-bactericidal activity, which involves chemistry at the bacterial level, in contrast to the purely mechano-bactericidal model currently proposed [46].

Furthermore, the species of bacteria as a biological factor is not of high importance in the ML model, a possible reason is the limited dataset, which focuses on only a few specific bacteria. Whereas it is now generally accepted that Gram-negative bacteria are more vulnerable to the bactericidal effects of nanostructures than Gram-positive bacteria because of the differences between their bacterial membrane structures. In the SHAP dependence analysis (Fig. 7c and d), we posit that Gram-positive bacteria demonstrate increased sensitivity to hydrophilic surfaces with nanostructured spacing below 250 nm. While the SHAP dependence plot distribution for Gram-negative bacteria in relation to WCA and spacing appears relatively dispersed.

#### Individual data points analysis and comparative analysis

To enhance the comprehension of why certain features exhibit a more pronounced impact than others within our dataset, we employed an analysis of individual SHAP value plots corresponding to specific data points. We selected three representative data points for this analysis, two of which are presented below, with the remaining details provided in Fig. S5 (Tables 5 and 6).

**Table 5:** Typical data points selected for the individual data points analysis

No	Material	Substrate						Bacteria				BE%	Ref
		Nano topography					WCA (°)	Strain	GS	Motility	Shape		
		Shape	Size(nm)			Aspect ratio							
			Diameter	Height	Spacing								
1	Si	pillar	21	212	50	0.10	8	P. aeruginosa	-	M	R	84%	[48]
2	Ti	tube	70	550	70	0.13	14	P. aeruginosa	-	M	R	25%	[49]
3	Si	pillar	70	680	180	0.10	0	R. capsulatus	-	M	R	50%	[30]

**Table 6:** Machine learning models frequently used in the biomedical field

Model	Synonym	Model Category	Description	Tuning Parameters
K-nearest neighbors	KNN	Simple model	Each feature value corresponds to a specific coordinate. The classification process consists of identifying the K nearest neighbours of a given data point and assigning it the most prevalent label among these neighbours [39].	K
Support vector machine	SVM	Support vector machine	In SVM, each data item is plot as a point in an n-dimensional space with the value of each feature related to the value of a specific coordinate. It performs classification by finding the hyper-plane that differentiates the two classes [41].	C
Stochastic Gradient Boosting	GBM	Ensemble model	By combining multiple weak learners, GBM creates a powerful ensemble model. In classification, it uses a specific loss function to process the classification results, providing high accuracy and flexibility [56].	n.trees; shrinkage; n.minobsinnode
eXtreme Gradient Boosting	XGBoost	Ensemble model	XGBoost is an optimised and enhanced version of GBM that enhances the gradient boosting algorithm by introducing regularisation, sparsity awareness, parallel learning, missing value handling and tree pruning [40].	max_depth; n_estimators; learning_rate; colsample_bytree…
Multilayer Perceptron	MLP	Feedforward artificial neural network	The MLP incorporates a series of hidden layers positioned between the input and output layers, forming a directly connected mechanism. This allows data to flow forward through the network, resembling a feed-forward network structure [42].	hidden_layer_size

##### Case 1: Silicon-based nano pillar against P. Aeruginosa

**Fig. 8:**
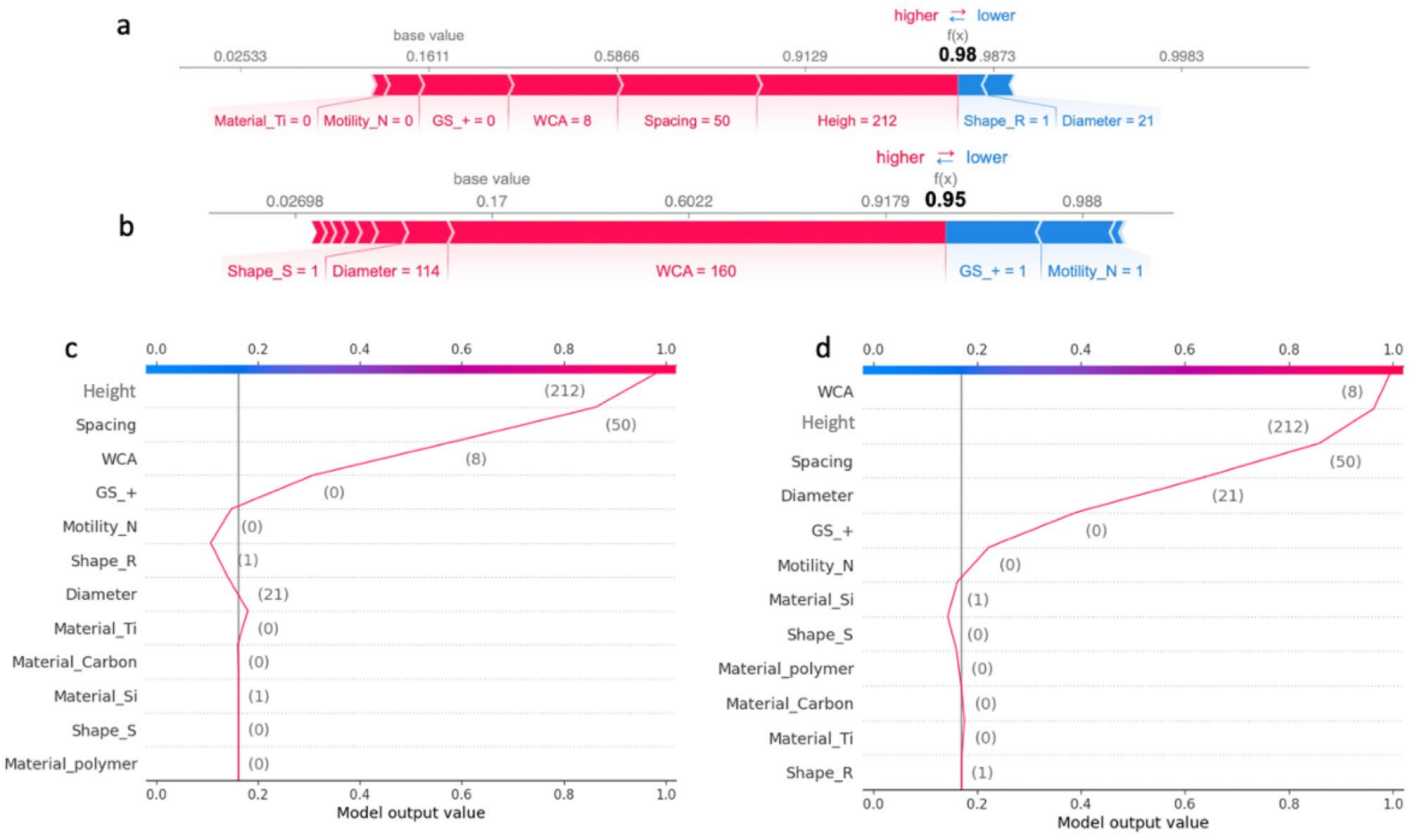
Comparative Analysis of Individual SHAP Values for the XGBoost-III Model and MLP-III Model - Case 1: (**a**) Individual SHAP force plot for XGBoost-III Model; (**b**) Individual SHAP force plot for MLP-III Model; (**c**) Individual SHAP decision plot for XGBoost-III Model; (**d**) Individual SHAP decision plot for MLP-III Model

Figure 8 illustrates that ‘Height’ has a significant positive SHAP value, indicating that as the height of the nanostructures increases, it contributes more to the model’s prediction of bactericidal efficiency against P.aeruginosa cells. This aligns with the conclusion in this study [12], which suggests that higher nanostructures on surfaces lead to a decrease in bacterial adhesion due to reduced contact area between the bacteria and the substratum.

In contrast, ‘Material’ has a minor impact on the output value, which is consistent with the previous reports stating that the nanoscale topography influences bacterial attachment behaviour, orientation, and the expression of attachment organelles (fimbriae), with a preference for certain substratum types [49].

The importance of height in these figures supports the notion that the physical dimensions of surface nanoarchitecture and material stiffness are critical factors in the adhesion and potential killing of bacterial cells.

##### Case 2: Titanium-based nano tube against P. Aeruginosa

**Fig. 9:**
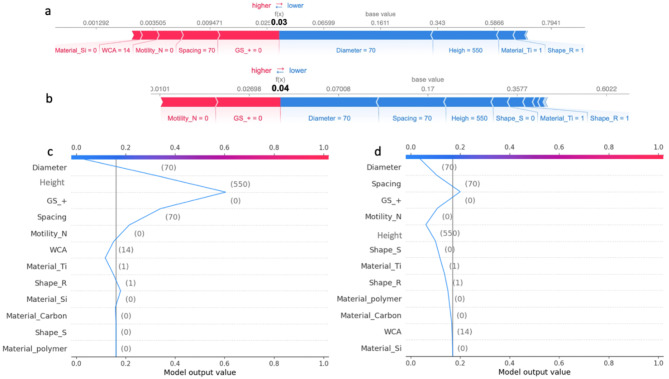
Comparative Analysis of Individual SHAP Values for the XGBoost-III Model and MLP-III Model - Case 2: (**a**) Individual SHAP force plot for XGBoost-III Model; (**b**) Individual SHAP force plot for MLP-III Model; (**c**) Individual SHAP decision plot for XGBoost-III Model; (**d**) Individual SHAP decision plot for MLP-III Model

In this case, the dimensions, specifically the diameter and height, of the nanostructures used in the dataset are significantly smaller relative to the overall range observed. In Fig. 9, although the ‘GS’ feature exerts a significant positive effect on the output value, the adverse impacts attributable to both ‘Diameter’ and ‘Height’ on the bactericidal effectiveness of the nanostructures culminate in a final model output of zero. The study that includes this case involved assessing the bactericidal efficiency of nanostructures with identical structural parameters against various bacterial strains. Notably, the nanostructures demonstrated enhanced effectiveness in eliminating Gram-positive bacteria.

Furthermore, the positive impact associated with ‘GS’ indicates that the model identifies the presence of Gram-negative bacteria as a factor reducing the likelihood of poor bactericidal performance, which is in alignment with the conclusion of the study [48]. While the SHAP value analysis for ‘WCA’, suggests a negligible role of this feature in bactericidal efficiency. The implication is that surfaces do not exhibit extreme hydrophilicity, therefore having a relatively minor impact. The insights from the model support the observation that sharp, elongated nanostructures can disrupt bacterial cells non-selectively, whereas shorter, blunt structures might necessitate more precise interactions to overcome the defences of different bacterial species, reflecting their adaptation to the ecological niches they inhabit [30].

In addition, we performed a comparison of the SHAP values for both the XGBoost and MLP algorithms by examining them in each case, as illustrated in the accompanying Figs. 8 and 9 and Fig. S4. The consistency of the results across these scenarios underscores the robustness and interpretative capability of our model.

### Regression model building

Based on the results of the classification model, a regression model was further developed for nanostructured surfaces with bactericidal efficiency greater than 70%. Figure 8 shows the distribution of bactericidal efficiency in the dataset and the range of data targeted by the classification/regression model.

**Fig. 10:**
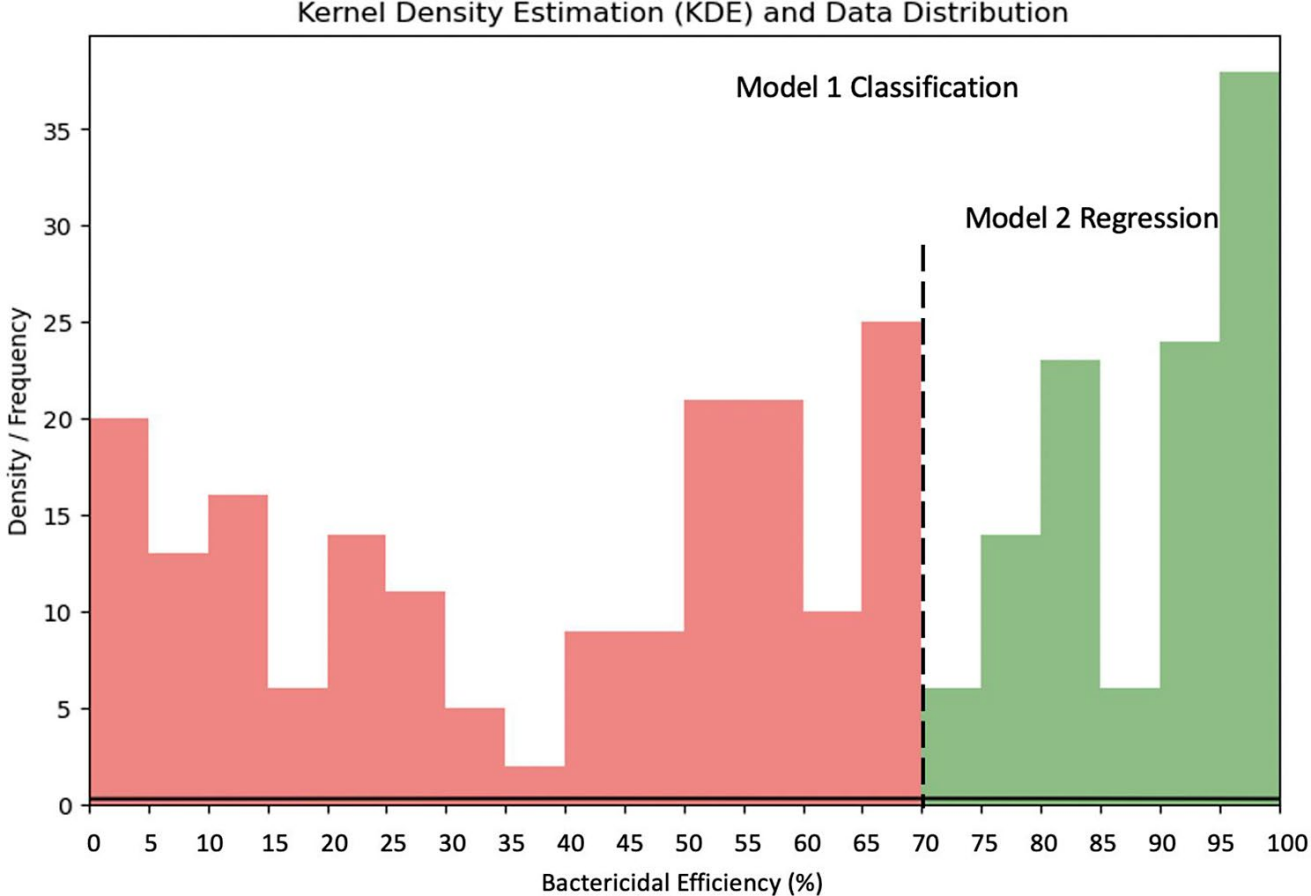
Sequence of Classification and Regression model that predicts bactericidal efficiency of nanostructured surface. The classification model determines whether the nanostructured surface is capable of effective bactericide, i.e., whether the bactericidal efficiency is greater than or equal to 70%. The regression model predicts values of bactericidal efficiency for nanostructured surfaces with > 70% bactericidal efficiency

**Fig. 11:**
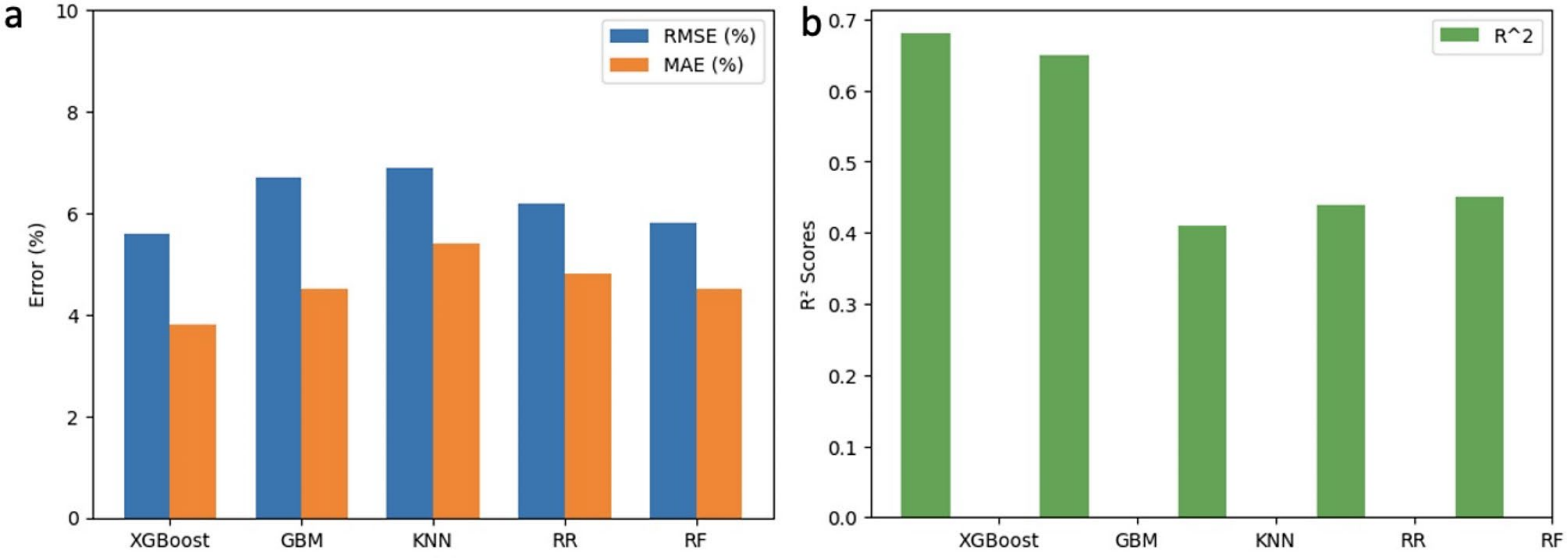
Regression model performance evaluated by (**a**) RMSE, MAE and (**b**) $\:{R}^{2}$

**Fig. 12:**
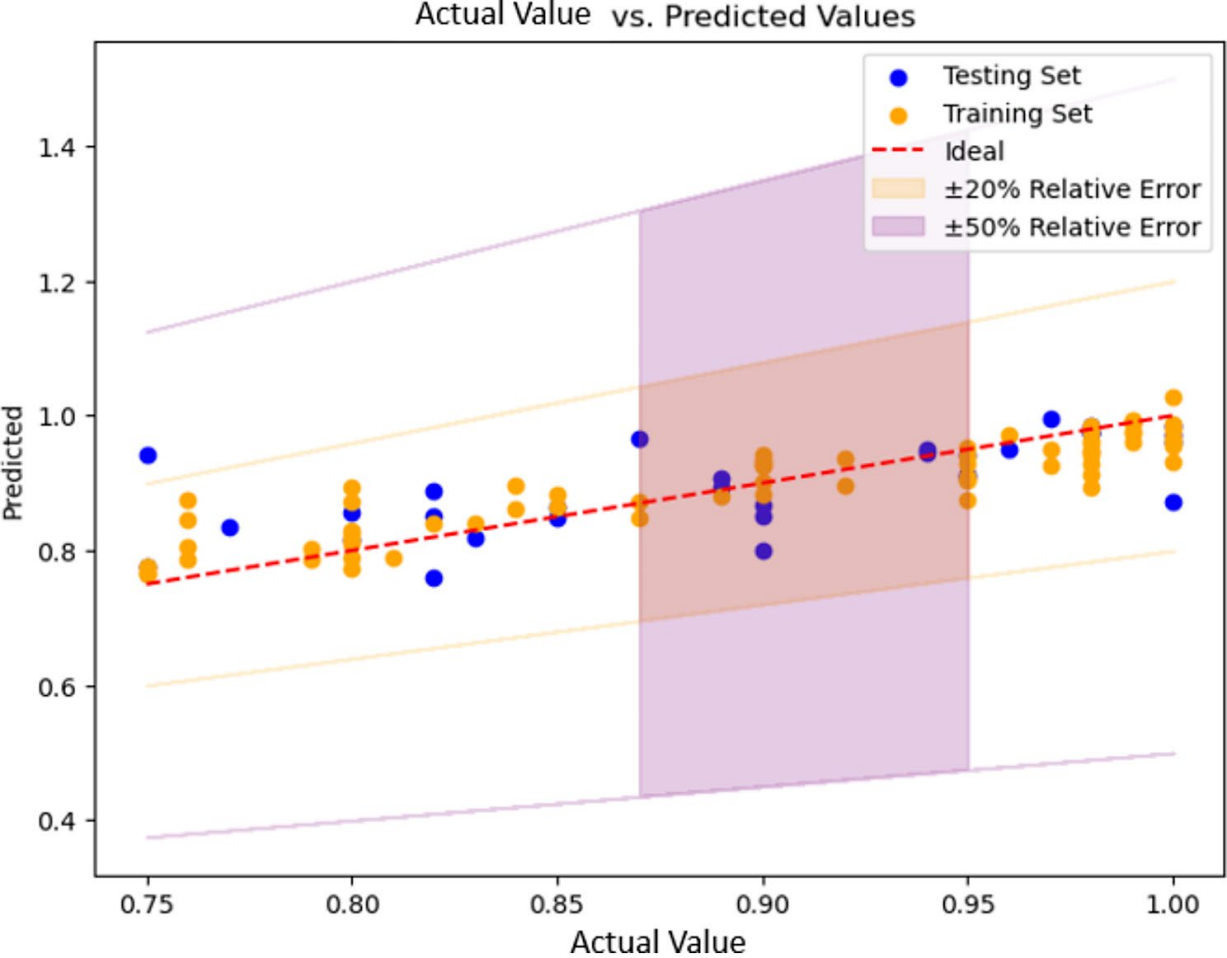
Predictions given by XGBoost model based on the data records in the Database IV. The red line shows perfect predictions where the ground truth values equal to predictions. The coloured area indicates the relative error range (± 20% and ± 50%) for the predictions

By traversing all the model parameters, the best combination of parameters is selected (see Table S2). The performance results are summarised in Fig. 9 and Table S4. As mentioned above, lower RMSE and MAE values indicate better predictive performance, while higher $\:{R}^{2}$ values indicate a better fit of the model to the data and a better overall adaptation to the data. Of the four models, the XGBoost regression model had an outstanding performance with the lowest RMSE and MAE and the highest $\:{R}^{2}$ (50%). The relatively low $\:{R}^{2}$ values observed in the table may be attributed to the limited amount of data available for analysis (Figs. 10, 11, and 12).

The regression model showed consistent performance on both the training and test sets, with all predictions within a relative error of ± 20%, except for one data from the test set (Fig. 10). This demonstrates the model’s ability to withstand overfitting trends and enhances its potential for real-world applications.

## Discussion

In this study, we implemented an ML model from data collection to model validation, to predict the bactericidal effects induced by nanostructured surface in in-vitro systems. We propose the concept of drawing patterns from existing studies on the bactericidal properties of nanostructured surface to guide future research. In the conventional materials research process, the selection of material candidates and topologies is entirely dependent on the developer’s intuition, and these materials need to be prepared and tested successively; this leads to inefficiencies, high error rates, and high costs [50]. Indeed, the further development of our model can help researchers in the field of nanostructured surfaces to save experimental resources and time costs. By analysing the feature importance derived from our model, researchers can gain insights into which parameters most significantly impact bactericidal efficiency. This can help in designing experiments that specifically target these influential factors, thereby optimizing the allocation of resources towards experiments that are most likely to yield significant findings. The predictive model can be used to generate hypotheses regarding the underlying mechanisms driving bactericidal effectiveness. For example, if the model identifies a specific nanotopography parameter as particularly influential, experimental work can be tailored to investigate how this parameter affects bacteria at the molecular or cellular level, leading to a deeper understanding of the interaction mechanisms.

As new materials are developed, our model provides a benchmark for predicting their potential bactericidal efficiency based on their properties and the bacteria type they are intended to target. This predictive capacity can streamline the initial screening process for novel materials, prioritizing those with the highest predicted efficacy for further experimental validation. Furthermore, by outlining the predictive relationships between various parameters and bactericidal efficiency, our model serves as a platform for fostering collaboration between materials scientists, microbiologists, and nanotechnology researchers. This interdisciplinary approach can lead to the development of more comprehensive experimental studies that consider a wider range of variables and their interactions.

The availability of data in materials science and other fields has long been a key issue, thus the recent concerted efforts have been focused on finding ML algorithms applicable to small datasets [51]. However, objectively comparing nanostructured surfaces with different parameters becomes more challenging due to the lack of standardized test methods to assess their performance [52]. While several valid methods may be available, provided they provide relevant information for the intended application, the lack of standard test conditions hinders direct comparisons between new techniques. Ideally, these standard conditions should be established and expanded to discover and verify the true effectiveness and utility of materials. Several ongoing initiatives are developing frameworks, methods and criteria for assessing the quality of reported data, based on the FAIR (Findable, Accessible, Interoperable, Reusable) data principles [53]. This may overcome the lack of data in the materials field and allow different studies to be compared more directly.

This research pragmatically proposes a different approach by developing prediction tools using well-established ML algorithms but comparing the impact of different data imputation methods on model performance. The rational data imputation method here allows us to apply the ML model to a larger dataset, thus improving the accuracy of the model.

Our dataset is drawn from a variety of published research, including different experimental setups being used by different research groups. This diversity of experimental setups enhances the generalization capabilities of our ML models.

The goal of our future work is to attempt to quantify the features of biological processes between nanostructured surfaces and bacteria in order to optimize the feature engineering of the ML model.

## Conclusion

In this study, we have successfully applied data extracted from the literature to build effective ML models to predict the antimicrobial properties of nanostructured surfaces, with gradient boosting machine (GBM) being the best model (The accuracy rate reached 81%). A regression model was further developed for nanostructured surfaces with bactericidal efficiency greater than 70% to further predict the value of bactericidal efficiency, with XGBoost being the best model. The feature importance analysis and the interpretation of the ML models suggest that the WCA is the most important feature, followed by nanotopography (e.g., diameter, height). We then analysed the interrelated features by calculating SHAP values to further understand the mechanism of mechano-bactericidal properties, and results showed that WCA, diameter, height and aspect ratio are positively correlated with bactericidal efficiency, while spacing is the opposite. We also highlight the need for standardized measurements to evaluate the antimicrobial properties of nanostructured surfaces, allowing more consistent metadata. Overall, this is the first study of applying ML algorithms in the field of nanostructured surface, which assists researchers in taking advantage of developments in the field of artificial intelligence, thereby improving timeliness, and reducing the number of experiments performed and the associated costs.

## Methods

### Data Collection

A literature search was conducted to identify studies examining the effects of nanostructured surfaces on bacterial elimination or inhibition. The collected articles were published up to March 2023, encompassed various keywords, including “antibacterial,” “antimicrobial,” “bactericidal,” “nanopattern,” “nanopatterned topography,” “nanostructure,” “nanostructured surface,” “nano-coating,” and more. The initial screening of articles was based on the title and abstract, followed by a secondary screening after thoroughly reading the full text.

#### Inclusion criteria

To be included in the analysis, studies had to meet both of the following requirements:

- Fabrication of nanoscale structures on surfaces.
- Conducting bacterial viability tests on these nanostructured surfaces.

#### Exclusion criteria

Studies were typically excluded from the analysis based on the following factors:

- Absence of bacterial viability quantification or unspecified method.
- Absence of surface topography quantification or unspecified method.
- Unrelated studies on the bactericidal activity of nanostructured surfaces.
- Presence of antibiotics or chemically active coatings on surfaces that contribute to bactericidal activity.
- Experiments are carried out under dynamic conditions, such as fluid flow, agitation, and so on.

### Data extraction

Each paper was reviewed with a focus on:

- Surface: materials, surface wettability, and surface roughness.
- Nanopattern: fabrication method, nanopattern tip dimension (width or diameter), and height, spacing.
- Bacteria viability: species, Gram-stain type, motility, and shape.

The above variables were acquired as input attributes for the prediction of the bactericidal efficiency of the investigated nanostructured surface. For the evaluation of bactericidal efficacy, we have comprehensively integrated quantitative evaluations derived from a diverse range of detection methods and techniques. The bacterial efficiency of most studies is calculated based on indicators such as bacterial viability, zone of inhibition (ZOI), and optical density (OD).

### Data pre-processing

#### Data imputation

Because our data is extracted from published research articles, inevitably it contains a considerable number of missing values due to different research strategies and incomplete reporting of parameters. Thus, five different strategies were implemented to handle the missing values.

- None: The None method directly deleted all records that contained missing values for comparison of performance.
- Leave empty: The leave_empty method left the records with missing values as ‘‘NaN” in the database without any processing.

The other three of them were actively filling the blank values through different data imputation techniques:

- Mean: The Mean method, as suggested by its name, used the mean value of that variable in the database to fill the blank.
- K-nearest neighbour (kNN): the kNN method imputed the missing values with the average of k-th most ‘‘similar” experiment.
- Random Forest (RF): The RF method, which built on random subsets of the available features and data samples, can provide accurate estimates for missing values by considering the information contained within the remaining variables [54].

#### One hot encoding

In numerous instances, algorithms encounter difficulties in effectively managing categorical values, as the majority of them necessitate numerical inputs to attain optimal outcomes. Consequently, in this research, the conversion of categorical variables, such as materials, bacterial motility, and bacterial shape, into numerical dummy variables or integers is essential.

ML algorithms frequently interpret the order of integers as a significant feature, which can pose challenges when addressing categorical data that lacks inherent relationships or rankings. In our situations where categorical values are devoid of a natural hierarchy, employing one-hot encoding proves advantageous to circumvent issues related to predictions and performance. To execute one-hot encoding, dummy variables were generated for each categorical column, and these new columns were subsequently integrated into the primary data frame. Utilizing binary values of 0 and 1, the absence or presence of the original attributes were denoted, respectively. This encoding method ensures that the categorical data can be effectively processed by machine learning algorithms without generating erroneous assumptions concerning the relationships among categories.

### Data split

For a supervised computational algorithm, a training set and a test set are initially provided. We randomly divide the data into two groups, one for training the model (training dataset) containing 70% of the dataset and the rest (30%) for testing performance (testing dataset).

### Classification model building and validation

The classification technique constructs a model with the ability to classify new instances into predefined categories based on the input variables. Classification modelling involves the task of learning a mapping function from inputs to discrete output categories. In this study, machine learning algorithms map the nanotopogrpahy of nanostructured surfaces and the functions of the targeted bacterial properties to their bactericidal activity, thus enabling prediction of the bactericidal efficiency. To develop our model and determine the most accurate predictions, we explored several supervised classification algorithms as potential candidates, including KNN, SVM, GBM, XGBoost, MLP, and RR. Models were built in Python version 3.9.13, Scikit-learn version 0.24.1.

#### Preliminary modelling

Since XGBoost can manage missing values in the input data, enabling it to learn from incomplete datasets, it allows for a fair and unbiased comparison of various imputation methods by evaluating their impact on the model’s performance. Thus, we choose XGBoost to do the comparison of different imputation strategies, and accuracy, precision, recall and F1-score are set as metrics to evaluate the model’s performance, i.e., to indicate how accurately the model classifies positive and negative samples. They were calculated by:

$$\:accuracy=\frac{(TP+TN)}{(TP+TN+FP+FN)}$$

$$\:precision=\frac{TP}{(TP+FP)}$$

$$\:recall=\frac{TP}{(TP+FN)}$$

$$\:F1\:Score=2\times\:\frac{(precision\times\:recall)}{(precisio+recall)}$$

where True Positives (TP) are instances where the model correctly predicts the positive class; True Negatives (TN) are instances where the model correctly predicts the negative class; False Positives (FP) are instances where the model has falsely identified a negative instance as positive, leading to a false alarm; False Negatives (FN) are instances where the model has failed to identify a positive instance, resulting in a missed detection.

Thereafter, 10-fold cross validation was applied to evaluate the stability of the models using different imputation methods. By using all available data for training and testing, k-fold cross-validation reduces the variance of the performance estimate compared to a simple train-test split. It also helps to avoid overfitting, as the model is evaluated on multiple independent test sets. Additionally, k-fold cross-validation can help to identify potential issues with the data, such as data leakage or class imbalance, that may not be apparent with a single train-test split. The dataset is split into k subsets or folds of equal size. The model was trained and evaluated k times, in each iteration, k-1 folds were used to train the model and the rest of the folds were used for testing. It can provide more reliable estimate of the model’s performance by mitigating the issues of overfitting and variability in the training and testing process.

#### Classification model building and validation

The data imputation method that gave the best results after preliminary modelling was used to build models with the post-imputation dataset and each of the five algorithms mentioned above. The accuracy, precision, recall, F1 scores and confusion matrixes of all models were compared. The performances of ML models were evaluated through 10-fold cross validation.

In order to test the predictive performance of the model and to provide guiding concepts for the design of nanostructured surfaces, four new datasets were created for prediction: Ti-based nanostructured surfaces against Gram-negative bacteria, Ti-based nanostructured surfaces against Gram-positive bacteria, Si-based nanostructured surfaces against Gram-positive bacteria and Si-based nanostructured surfaces against Gram-negative bacteria. Each dataset contained 829,448 data points, from which the bactericidal efficiencies were plotted. The enumeration parameters are detailed in Table 4.

### Analysis of feature importance

For all ML models, the feature importance is calculated be using Permutation Importance algorithm, which works by first training the model on the original dataset and calculating the performance metric on a validation set. Then, for each feature, the values of that feature in the validation set are randomly permuted (shuffled) while leaving the values of all other features unchanged. The model is then evaluated on the permuted data, and the difference between the original performance and the permuted performance is calculated. This difference represents the importance of the feature.

For GBM and XGBoost model, the feature importance is also calculated based on the reduction in the loss function that is achieved by a feature when it is used in a decision tree for comparison. For each feature, the importance score is calculated by summing the gain in accuracy or reduction in the objective function that was achieved when using that feature for splitting data over all the decision trees in the model. The feature importance scores are normalized so that they sum to 1.

Despite identifying the important features that contributed to bactericidal efficiency prediction, the extent of the contributions is unknown. This is a typical characteristic of models generated with most ML methods, as interpreting the predictive model’s output is tedious, especially by increasing the complexities in non-linear models. Thus, we used SHAP values to explain how features affect the output of ML models. This framework has a solid theoretical foundation in game theory and can provide contrastive explanations and analyse the model’s output locally and globally [56].

### Regression model building

In this study, after successfully constructing a classification model, a regression model was built to extend the analysis by using four different algorithms to further analyse the bactericidal efficiency of successful bactericidal surfaces: KNN, GBM, XGBoost and Ridge Regression (RR).

RR has been added, a linear regression regularisation technique that adds a penalty term to the loss function to help prevent overfitting by reducing the coefficients to zero. It is particularly useful in cases where there is multicollinearity, providing a balance between the complexity of the model and generalisation performance.

To evaluate the models’ performances, Root Mean Squared Error (RMSE), mean absolute error (MAE), and coefficient of determination ($\:{R}^{2}$) were set as the metrics. They were calculated by:

$$\:RMSE=\:\sqrt{\frac{\sum\:_{i=1}^{N}{({y}_{i}-\widehat{{y}_{i}})}^{2}}{N}}$$

$$\:MAE=\:\frac{1}{N}\sum\:_{i=1}^{N}\left|{y}_{i}\right.-\left.\widehat{{y}_{i}}\right|$$

$$\:{R}^{2}=\:\sqrt{\frac{\sum\:_{i=1}^{N}{(\stackrel{-}{{y}_{i}}-\widehat{{y}_{i}})}^{2}}{\sum\:_{i=1}^{N}{(\stackrel{-}{{y}_{i}}-{y}_{i})}^{2}}}$$

where $\:{y}_{i}$ is the i’th expected value in the dataset, $\:\widehat{{y}_{i}\:}\:$is the i’th predicted value, and $\:\stackrel{-}{{y}_{i}}$ is the mean of expected value.

The effectiveness of a predictive model is often gauged by its RMSE and MAE values, which are relative to the specific dataset used. Generally, smaller values indicate a more accurate model. $\:{R}^{2}$ is a statistical measure of fit that suggests how much variation of the output is supported by the inputs. $\:{R}^{2}$ explains to what degree the variance of one variable describes the variance of the second variable.

## Electronic supplementary material

Below is the link to the electronic supplementary material.

Supplementary Material 1

## Data Availability

No datasets were generated or analysed during the current study.
