# Supplementary material for: A supervised machine learning tool to predict the bactericidal efficiency of nanostructured surface
Source: J Nanobiotechnology. 2024 Dec 3;22:748. doi: 10.1186/s12951-024-02974-8 (PMC11613743; doi:10.1186/s12951-024-02974-8)
Supplement: Supplementary file 1 — Supplementary Material 1 [file 12951_2024_2974_MOESM1_ESM.docx]

**A Supervised Machine Learning Tool to Predict the Bactericidal Efficiency of Nanostructured Surface**

Yaxi Chen^1^, Hongyi Chen^2^, Anthony Harker^3^, Yuanchang Liu^1^, and Jie Huang^1^

1.Department of Mechanical Engineering, University College London, London, UK

2.Department of Computer Science, University College London, London, UK

3.Department of Physics & Astronomy, University College London, London, UK


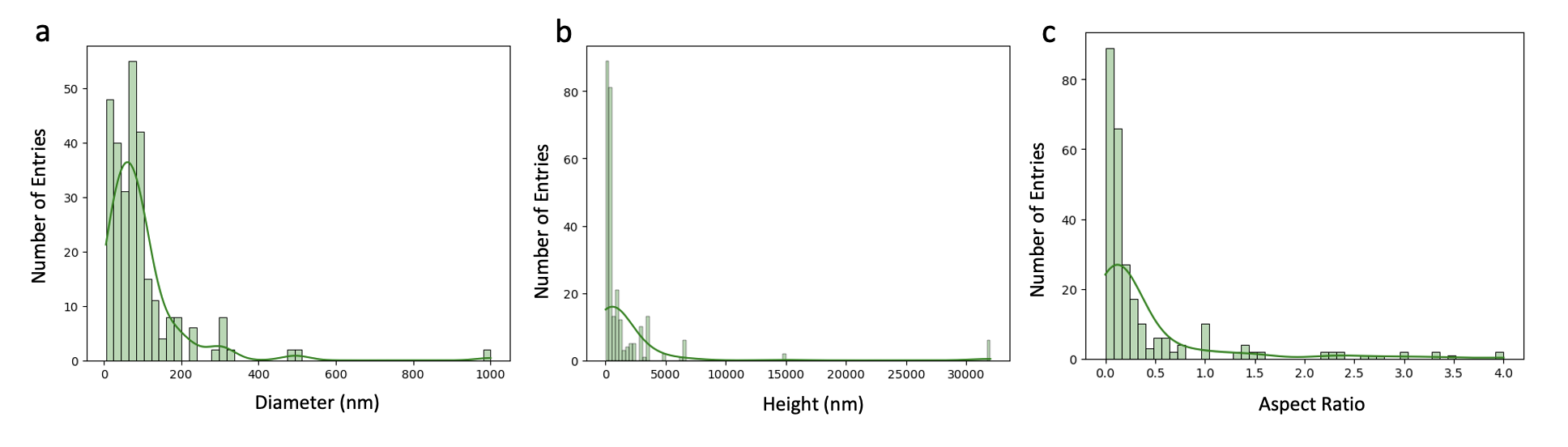


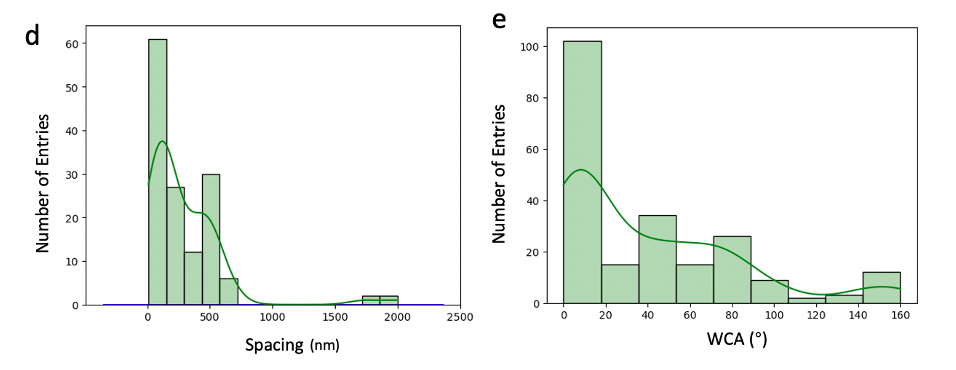


**Figure.S1** Data distributions of (a) Diameter, (b) Height, (c) Spacing, (d) Aspect Ratio, and (e) WCA.


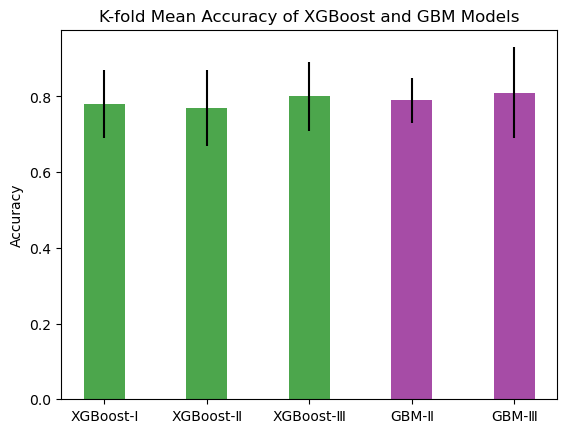


**Figure.S2** Classification model performance evaluated by mean accuracy obtained from 10-fold cross-validation. Error bars obtained from 10-fold CV.


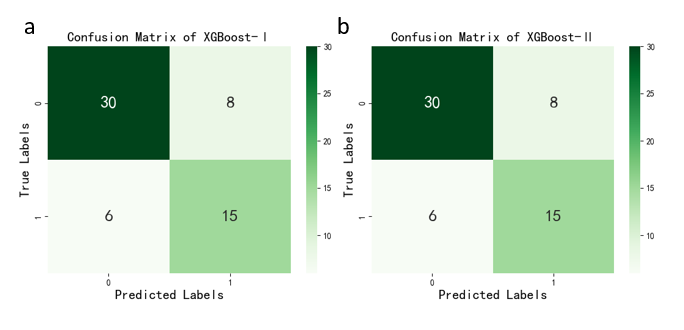


**Figure.S3** Confusion matrix of (a) XGBoost-Ⅰ, (b) XGBoost-Ⅱ model (Label 0 for bactericidal efficiency <70%, Label 1 for bactericidal efficiency ≥70%)


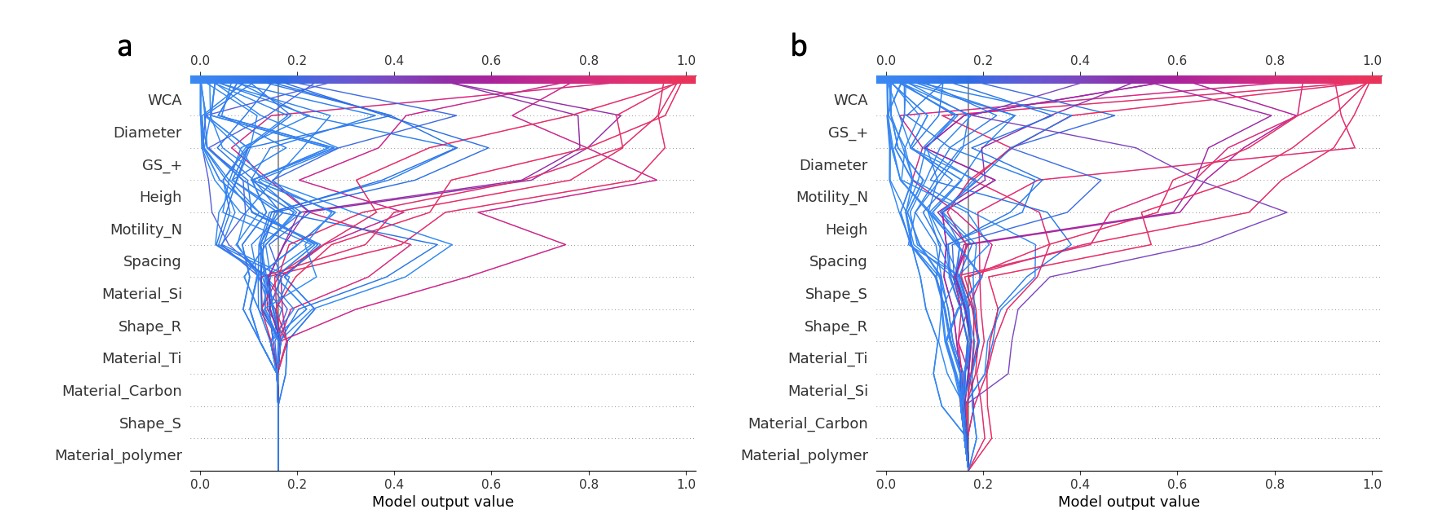


**Figure. S4** Summary decision plots for (a) XGBoost-III Model; (b) MLP-III Model. Each line represents a feature and its impact across all the samples in the dataset. The blue lines indicate features that push the model output lower, while the red lines indicate features that push the model output higher. Features at the top have a more positive impact, and features at the bottom have a more negative impact.

**Case 3: Silicon-based nano pillar against R. capsulatus**


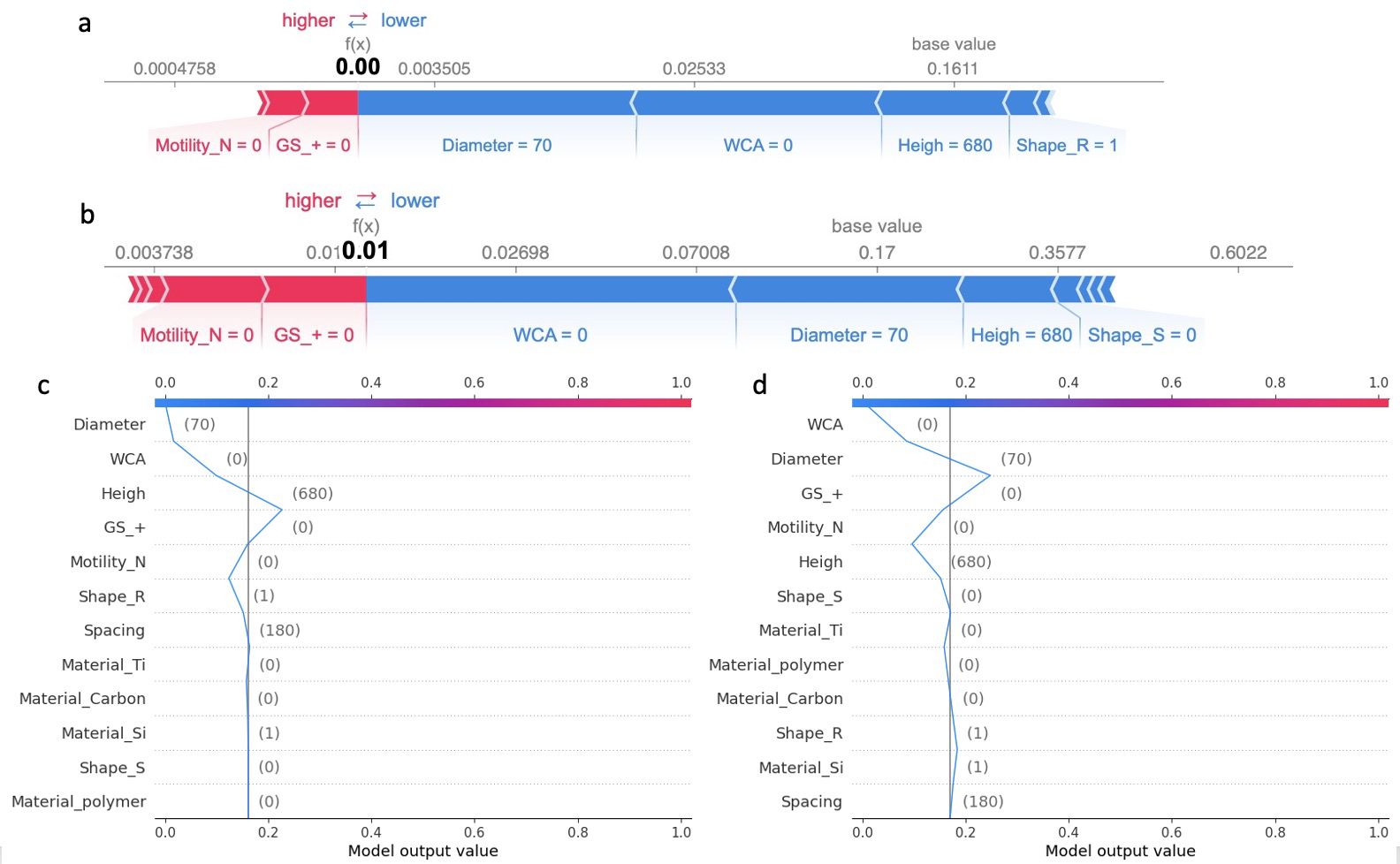


**Figure. S5** Comparative Analysis of Individual SHAP Values for the XGBoost-III Model and MLP-III Model - Case 3: (a) Individual SHAP force plot for XGBoost-III Model; (b) Individual SHAP force plot for MLP-III Model; (c) Individual SHAP decision plot for XGBoost-III Model; (d) Individual SHAP decision plot for MLP-III Model

In this case, the nanostructure presents a smaller height and diameter compared to other structures within the same study. The height, recorded at 680 nm, significantly impacts the model's output negatively. This suggests that the physical height of the nanostructures is crucial, with taller structures potentially offering greater efficiency in penetrating the microbial cell walls across different species. In contrast, the diameter, measured at 70 nm, also negatively affects the model's performance but to a lesser degree. This implies that the diameter influences the interaction between the nanostructure's surface and bacterial cells, where larger diameters might enhance the surface's ability to stretch and disrupt the cell walls of various bacterial species.

Furthermore, the material features of the nanostructures were found to have no impact on the model's output for this data point, indicating that the antibacterial effectiveness might not be significantly altered by the material composition. Instead, the physical attributes of the nanostructures, such as height, diameter, and spacing, appear to play a more critical role in determining the outcome.

**Table.S1** Optimized hyperparameters of ML classification models

| Model | Hyperparameters |
| --- | --- |
| kNN | n_neighbors=5 |
| SVM | C = 10, kernel=’rbf’, gamma=0.8 |
| MLP | Number of Hidden Layers: 2; hidden_layer_size=(100, 100); Activation Function: ReLU; Solver for Weight Optimization: Adam; Regularization (Alpha): 0.0001; Learning Rate: Constant with an initial value of 0.001  Maximum Iterations: 200 |
| XGBoost | learning_rate=0.1, subsample=1, reg_lambda=1.1, n_estimators=100, max_depth=8, colsample_bytree=0.5, gamma=0.5, min_child_weight=1, max_delta_step=0, colsample_bytree=0.7, reg_alpha=0.1, reg_lambda=0.5 |
| GBM | learning_rate=0.2, subsample=0.8, max_depth=5 |

**Table.S2** Optimized hyperparameters of ML regression models

| Model | Hyperparameters |
| --- | --- |
| kNN | n_neighbors=4 |
| XGBoost | learning_rate=0.01, subsample=1, reg_lambda=1.1, n_estimators=1000, max_depth=3, colsample_bytree=0.5, gamma=0.5, min_child_weight=6, max_delta_step=0, colsample_bytree=0.7, reg_alpha=0.1, reg_lambda=0.4 |
| GBM | learning_rate=0.01, subsample=1, max_depth=3, n_estimators=100 |
| RR | Alpha=1.0, ma_iter=1000, tol=0.001 |

**Table.S3** A comparison of the performances of the classification models and validation by 10-fold cross validation

| N | Dataset | Algorithm | Model Performance | | | | Cross-validation |
| --- | --- | --- | --- | --- | --- | --- | --- |
|  |  |  | Accuracy | Precision | Recall | F1 |  |
| 1 | Ⅰ | XGBoost | 0.76 | 0.66 | 0.68 | 0.67 | 0.78 ± 0.09 |
| 2 | Ⅱ | XGBoost | 0.78 | 0.69 | 0.65 | 0.67 | 0.77 ± 0.10 |
| 3 | Ⅲ | XGBoost | 0.76 | 1.00 | 0.41 | 0.58 | 0.80 ± 0.09 |
| 4 | Ⅱ | KNN | 0.73 | 0.83 | 0.44 | 0.57 | 0.68 ± 0.13 |
| 5 | Ⅲ | KNN | 0.71 | 0.71 | 0.46 | 0.56 | 0.70 ± 0.12 |
| 6 | Ⅱ | SVM | 0.62 | 0.50 | 0.14 | 0.21 | 0.69 ± 0.07 |
| 7 | Ⅲ | SVM | 0.71 | 0.50 | 0.25 | 0.33 | 0.73 ± 0.06 |
| 8 | Ⅱ | MLP | 0.62 | 0.50 | 0.36 | 0.42 | 0.62 ± 0.15 |
| 9 | Ⅲ | MLP | 0.71 | 0.80 | 0.36 | 0.50 | 0.65 ± 0.15 |
| 10 | Ⅱ | GBM | 0.76 | 0.69 | 0.75 | 0.72 | 0.79 ± 0.06 |
| 11 | Ⅲ | GBM | 0.93 | 0.91 | 0.91 | 0.91 | 0.81 ± 0.12 |
| 12 | Ⅱ | RF | 0.75 | 0.79 | 0.50 | 0.61 | 0.72 ± 0.06 |
| 13 | Ⅲ | RF | 0.75 | 0.85 | 0.48 | 0.61 | 0.74 ± 0.12 |

**Table.S4** Regression models performance evaluated by RMSE, MAE, and $R^{2}$

| **N** | **Dataset** | **Algorithm** | **Model Performance** | | |
| --- | --- | --- | --- | --- | --- |
|  |  |  | **RMSE** | **MAE** | $\boldsymbol{R}^{\mathbf{2}}$ |
| **13** | Ⅳ | XGBoost | 5.6% | 3.8% | 0.68 |
| **14** | Ⅳ | GBM | 6.7% | 4.5% | 0.65 |
| **15** | Ⅳ | KNN | 6.9% | 5.4% | 0.41 |
| **16** | Ⅳ | RR | 6.2% | 4.8% | 0.44 |
| **17** | Ⅳ | RF | 5.8% | 4.5% | 0.45 |

**Table S5.** Raw dataset of nanostructured surface and its antimicrobial properties

| Material | Fabrication method | Substrate | | | | | | | | Bacteria | | | | BE% | Ref |
| --- | --- | --- | --- | --- | --- | --- | --- | --- | --- | --- | --- | --- | --- | --- | --- |
|  |  | Nano topography | | | | | surface roughness (nm) | | WCA (°) | Strain | GS | Motility | Shape |  |  |
|  |  | Shape | Size | | | Aspect ratio |  |  |  |  |  |  |  |  |  |
|  |  |  | Diameter/Width（nm） | Heigh（nm） | Spacing |  | Ra-Arithmetical mean height | Rq-root mean square height |  |  |  |  |  |  |  |
| Ti | Reactive Ion Etching (RIE) | pillar | 93 | 1510 | 503 | 0.06 | 202 | 246 | 55 | P. aeruginosa | - | M | R | 32% | (1) |
| Ti | Reactive Ion Etching (RIE) | pillar | 93 | 1510 | 503 | 0.06 | 202 | 246 | 55 | S. aureus | + | N | S | 12% |  |
| Ti | Reactive Ion Etching (RIE) | pillar | 103 | 1900 | 451 | 0.05 | 258 | 320 | 83 | P. aeruginosa | - | M | R | 68% |  |
| Ti | Reactive Ion Etching (RIE) | pillar | 103 | 1900 | 451 | 0.05 | 258 | 320 | 83 | S. aureus | + | N | S | 37% |  |
| Ti | Reactive Ion Etching (RIE) | pillar | 115 | 2100 | 688 | 0.05 | 322 | 405 | 96 | P. aeruginosa | - | M | R | 68% |  |
| Ti | Reactive Ion Etching (RIE) | pillar | 115 | 2100 | 688 | 0.05 | 322 | 405 | 96 | S. aureus | + | N | S | 4% |  |
| Ti | Reactive Ion Etching (RIE) | pillar | 135 | 2300 | 593 | 0.06 | 286 | 351 | 111 | P. aeruginosa | - | M | R | 87% |  |
| Ti | Reactive Ion Etching (RIE) | pillar | 135 | 2300 | 593 | 0.06 | 286 | 351 | 111 | S. aureus | + | N | S | 15% |  |
| Ti | Reactive Ion Etching (RIE) | pillar | 158 | 3500 | 1730 | 0.05 | 446 | 567 | 98 | P. aeruginosa | - | M | R | 76% |  |
| Ti | Reactive Ion Etching (RIE) | pillar | 158 | 3500 | 1730 | 0.05 | 446 | 567 | 98 | S. aureus | + | N | S | 73% |  |
| Ti | Reactive Ion Etching (RIE) | pillar |  | 500 |  |  | 57 | 72 | 18 | E.coli | - | M | R | 80% | (2) |
| Ti | Reactive Ion Etching (RIE) | pillar | 80 | 1000 |  | 0.08 | 186 | 227 | 10 | E.coli | - | M | R | 95% |  |
| Ti | Reactive Ion Etching (RIE) | pillar | 80 | 1000 |  | 0.08 | 186 | 227 | 10 | P. aeruginosa | - | M | R | 98% |  |
| Ti | Reactive Ion Etching (RIE) | pillar | 80 | 1000 |  | 0.08 | 186 | 227 | 10 | S. aureus | + | N | S | 22% |  |
| Ti | Reactive Ion Etching (RIE) | pillar | 80 | 1000 |  | 0.08 | 186 | 227 | 10 | S. aureus(24h) | + | N | S | 76% |  |
| Ti | Reactive Ion Etching (RIE) | pillar | 80 | 1000 |  | 0.08 | 186 | 227 | 10 | M. smegamatis | + | M | R | 92% |  |
| Ti | Reactive Ion Etching (RIE) | pillar |  | 2000 |  |  | 215 | 269 | 9 | E.coli | - | M | R | 95% |  |
| Ti | Hydrothermal synthesis | wire | 52 | 298 |  | 0.17 |  |  |  | S. aureus | + | N | S | 54% | (3) |
| TiO_2_ | Hydrothermal synthesis | wire | 1000 | 3000 |  | 0.33 |  |  |  | P. aeruginosa | - | M | R | 40% | (4) |
| TiO_2_ | Hydrothermal synthesis | wire | 1000 | 3000 |  | 0.33 |  |  |  | B. subtilis | + | M | R | 50% |  |
| TiO_2_ | Hydrothermal synthesis | wire | 100 | 3000 |  | 0.03 |  |  |  | S. aureus | + | N | S | 0% |  |
| TiO_2_ | Hydrothermal synthesis | wire | 100 | 3000 |  | 0.03 |  |  |  | E.coli | - | M | R | 80% |  |
| TiO_2_ | Hydrothermal synthesis | wire | 100 | 3000 |  | 0.03 |  |  |  | E. faecalis | + | M | S | 0% |  |
| TiO_2_ | Hydrothermal synthesis | wire | 100 | 3000 |  | 0.03 |  |  |  | K. pneumoniae | - | N | R | 5% |  |
| TiO_2_ | Hydrothermal synthesis | wire | 42 | 307 |  | 0.14 | 40 |  | 14 | S. aureus | + | N | S | 54% | (5) |
| TiO_2_ | Hydrothermal synthesis | wire | 42 | 307 |  | 0.14 | 40 |  | 14 | P. aeruginosa | - | M | R | 25% |  |
| TiO_2_ | Hydrothermal synthesis | wire | 33 | 1316 |  | 0.03 | 140 |  | 9 | S. aureus | + | N | S | 25% |  |
| TiO_2_ | Hydrothermal synthesis | wire | 33 | 1316 |  | 0.03 | 140 |  | 9 | P. aeruginosa | - | M | R | 0% |  |
| TiO_2_ | Hydrothermal synthesis | wire | 17 | 180 |  | 0.09 | 149 |  | 36 | S. aureus | + | N | S | 45% |  |
| TiO_2_ | Hydrothermal synthesis | wire | 17 | 180 |  | 0.09 | 149 |  | 36 | P. aeruginosa | - | M | R | 0% |  |
| TiO_2_ | Hydrothermal synthesis | wire | 18 | 328 |  | 0.05 | 27 |  | 7 | S. aureus | + | N | S | 20% |  |
| TiO_2_ | Hydrothermal synthesis | wire | 18 | 328 |  | 0.05 | 27 |  | 7 | P. aeruginosa | - | M | R | 0% |  |
| TiO_2_ | Hydrothermal synthesis | wire | 21 | 244 |  | 0.09 | 51 |  | 91 | S. aureus | + | N | S | 20% |  |
| TiO_2_ | Hydrothermal synthesis | wire | 21 | 244 |  | 0.09 | 51 |  | 91 | P. aeruginosa | - | M | R | 0% |  |
| TiO_2_ | Hydrothermal synthesis | wire | 154 | 14796 |  | 0.01 | 2296 |  | 6 | S. aureus | + | N | S | 25% |  |
| TiO_2_ | Hydrothermal synthesis | wire | 154 | 14796 |  | 0.01 | 2296 |  | 6 | P. aeruginosa | - | M | R | 0% |  |
| Ti | Hydrothermal synthesis | wire | 30 | 40 |  | 0.75 | 401 | 481 | 73 | P. aeruginosa | - | M | R | 47% | (6) |
| Ti | Hydrothermal synthesis | wire | 30 | 40 |  | 0.75 | 401 | 481 | 73 | S. aureus | + | N | S | 19% |  |
| Ti | Glancing angle sputter deposition (GLAD) | pillar | 33 | 478 | 158 | 0.07 |  | 26 | 41 | E.coli | - | M | R | 60% | (7) |
| Ti | Glancing angle sputter deposition (GLAD) | pillar | 33 | 478 | 158 | 0.07 |  | 26 | 41 | S. aureus | + | N | S | 0% |  |
| Ti | Glancing angle sputter deposition (GLAD) | pillar | 50 | 300 | 150 | 0.17 |  | 57 | 102 | S. aureus | + | N | S | 80% | (8) |
| TiO_2_ | Electrochemical  anodizing (EA) | pillar | 10 | 2000 | 2000 | 0.01 |  |  | 0 | S. aureus | + | N | S | 15% | (9) |
| TiO_2_ | Electrochemical  anodizing (EA) | pore | 55 | 1000 | 70 | 0.06 |  |  | 15 | S. aureus | + | N | S | 23% |  |
| Ti | Anodization | tube | 20 | 200 |  | 0.10 |  | 10 | 7 | S. aureus | + | N | S | 51% | (10) |
| Ti | Anodization | tube | 20 | 200 |  | 0.10 |  | 10 | 7 | S. epidermidis | + | N | S | 40% |  |
| Ti | Anodization | tube | 40 | 1200 |  | 0.03 |  | 8 | 7 | S. aureus | + | N | S | 52% |  |
| Ti | Anodization | tube | 40 | 1200 |  | 0.03 |  | 8 | 7 | S. epidermidis | + | N | S | 46% |  |
| Ti | Anodization | tube | 60 | 3600 |  | 0.02 |  | 10 | 7 | S. aureus | + | N | S | 54% |  |
| Ti | Anodization | tube | 60 | 3600 |  | 0.02 |  | 10 | 7 | S. epidermidis | + | N | S | 47% |  |
| Ti | Anodization | tube | 80 | 6500 |  | 0.01 |  | 9 | 9 | S. aureus | + | N | S | 58% |  |
| Ti | Anodization | tube | 80 | 6500 |  | 0.01 |  | 9 | 9 | S. epidermidis | + | N | S | 52% |  |
| Ti | Anodization + heat treatment | tube | 20 | 200 |  | 0.10 |  | 10 | 1 | S. aureus | + | N | S | 52% |  |
| Ti | Anodization + heat treatment | tube | 20 | 200 |  | 0.10 |  | 10 | 1 | S. epidermidis | + | N | S | 43% |  |
| Ti | Anodization + heat treatment | tube | 40 | 1200 |  | 0.03 |  | 8 | 2 | S. aureus | + | N | S | 60% |  |
| Ti | Anodization + heat treatment | tube | 40 | 1200 |  | 0.03 |  | 8 | 2 | S. epidermidis | + | N | S | 48% |  |
| Ti | Anodization + heat treatment | tube | 60 | 3600 |  | 0.02 |  | 10 | 2 | S. aureus | + | N | S | 49% |  |
| Ti | Anodization + heat treatment | tube | 60 | 3600 |  | 0.02 |  | 10 | 2 | S. epidermidis | + | N | S | 48% |  |
| Ti | Anodization + heat treatment | tube | 80 | 6500 |  | 0.01 |  | 9 | 3 | S. aureus | + | N | S | 51% |  |
| Ti | Anodization + heat treatment | tube | 80 | 6500 |  | 0.01 |  | 9 | 3 | S. epidermidis | + | N | S | 47% |  |
| Ti6Al4V | hydrothermally etched | cone | 90 | 348 | 437 | 0.26 | 500 |  | 61 | S. aureus | + | N | S | 65% | (11) |
| Ti6Al4V | hydrothermally etched | cone | 90 | 348 | 437 | 0.26 | 500 |  | 61 | P. aeruginosa | - | M | R | 98% |  |
| Ti6Al4V | hydrothermally etched | cone | 90 | 348 | 437 | 0.26 | 500 |  | 61 | P. aeruginosa | - | M | R | 90% |  |
| Ti+nano-CeO_2_ | hydrothermal methods | rod | 9.6 | 325 |  | 0.03 |  |  | 38 | S. sanguinis | + | M | S | 60% | (12) |
| Ti+nano-CeO_2_ | hydrothermal methods | rod | 9.6 | 325 |  | 0.03 |  |  | 38 | F. nucleatum | - | N | R | 0% |  |
| Ti+nano-CeO_2_ | hydrothermal methods | cube | 57.2 | 57.2 |  | 1.00 |  |  | 38 | S. sanguinis | + | M | S | 90% |  |
| Ti+nano-CeO_2_ | hydrothermal methods | cube | 57.2 | 57.2 |  | 1.00 |  |  | 38 | F. nucleatum | - | N | R | 0% |  |
| Ti+nano-CeO_2_ | hydrothermal methods | octahedra | 29.1 | 29.1 |  | 1.00 |  |  | 38 | S. sanguinis | + | M | S | 90% |  |
| Ti+nano-CeO_2_ | hydrothermal methods | octahedra | 29.1 | 29.1 |  | 1.00 |  |  | 38 | F. nucleatum | - | N | R | 0% |  |
| Ti | Hydrothermal etching | pore | 22.2 | 55.5 | 95.9 | 0.40 |  | 96.9 |  | S. aureus | + | N | S | 60% | (13) |
| Ti | Hydrothermal etching | pore | 22.2 | 55.5 | 95.9 | 0.40 |  | 96.9 |  | C.auris | - | N | S | 64% |  |
| Ti | Hydrothermal etching | pore | 20.8 | 75.4 | 94.5 | 0.28 |  | 84.2 |  | S. aureus | + | N | S | 64% |  |
| Ti | Hydrothermal etching | pore | 20.8 | 75.4 | 94.5 | 0.28 |  | 84.2 |  | C.auris | - | N | S | 70% |  |
| Ti | Hydrothermal etching | pore | 24.2 | 106.4 | 140.1 | 0.23 |  | 82.4 |  | S. aureus | + | N | S | 64% |  |
| Ti | Hydrothermal etching | pore | 24.2 | 106.4 | 140.1 | 0.23 |  | 82.4 |  | C.auris | - | N | S | 72% |  |
| Ti | Hydrothermal etching | pore | 40.3 | 210.1 | 193.7 | 0.19 |  | 104.6 |  | S. aureus | + | N | S | 70% |  |
| Ti | Hydrothermal etching | pore | 40.3 | 210.1 | 193.7 | 0.19 |  | 104.6 |  | C.auris | - | N | S | 70% |  |
| Ti | Reactive Ion Etching (RIE) | tube | 22 | 1480 | 10 | 0.01 | 32.89 | 43.87 | 24 | S. aureus | + | N | S | 42% | (14) |
| Ti | Reactive Ion Etching (RIE) | tube | 53 | 3520 | 16 | 0.02 | 53.6 | 68.83 | 10 | S. aureus | + | N | S | 40% |  |
| Ti | Reactive Ion Etching (RIE) | tube | 97 | 6200 | 25 | 0.02 | 82.53 | 110.67 | 7 | S. aureus | + | N | S | 50% |  |
| Si | Reactive Ion Etching (RIE) | pillar | 63 | 280 | 62 | 0.23 | 10 |  | 8 | P. aeruginosa | - | M | R | 89% | (15) |
| Si | Reactive Ion Etching (RIE) | pillar | 63 | 280 | 62 | 0.23 | 10 |  | 8 | S. aureus | + | N | S | 85% |  |
| Si | Reactive Ion Etching (RIE) | pillar | 80 | 430 | 100 | 0.19 | 29 |  | 12 | P. aeruginosa | - | M | R | 70% |  |
| Si | Reactive Ion Etching (RIE) | pillar | 80 | 430 | 100 | 0.19 | 29 |  | 12 | S. aureus | + | N | S | 65% |  |
| Si | Reactive Ion Etching (RIE) | pillar | 93 | 612 | 140 | 0.15 | 66 |  | 10 | P. aeruginosa | - | M | R | 82% |  |
| Si | Reactive Ion Etching (RIE) | pillar | 93 | 612 | 140 | 0.15 | 66 |  | 10 | S. aureus | + | N | S | 74% |  |
| Si | Reactive Ion Etching (RIE) | pillar | 50 | 500 |  | 0.10 |  |  | 0.8 | P. aeruginosa | - | M | R | 83% | (16) |
| Si | Reactive Ion Etching (RIE) | pillar | 50 | 500 |  | 0.10 |  |  | 0.8 | S. aureus | + | N | S | 53% |  |
| Si | Reactive Ion Etching (RIE) | pillar | 50 | 500 |  | 0.10 |  |  | 0.8 | B. subtilis | + | M | R | 90% |  |
| Si | Reactive Ion Etching (RIE) | pillar | 50 | 390 | 130 | 0.13 |  |  | 0 | B. subtilis | + | M | R | 65% | (17) |
| Si | Reactive Ion Etching (RIE) | pillar | 70 | 680 | 180 | 0.10 |  |  | 0 | B. subtilis | + | M | R | 75% |  |
| Si | Reactive Ion Etching (RIE) | pillar | 220 | 3580 | 380 | 0.06 |  |  | 0 | B. subtilis | + | M | R | 80% |  |
| Si | Reactive Ion Etching (RIE) | pillar | 50 | 390 | 130 | 0.13 |  |  | 0 | P. fluorescens | - | M | R | 60% |  |
| Si | Reactive Ion Etching (RIE) | pillar | 70 | 680 | 180 | 0.10 |  |  | 0 | P. fluorescens | - | M | R | 70% |  |
| Si | Reactive Ion Etching (RIE) | pillar | 220 | 3580 | 380 | 0.06 |  |  | 0 | P. fluorescens | - | M | R | 75% |  |
| Si | Reactive Ion Etching (RIE) | pillar | 50 | 390 | 130 | 0.13 |  |  | 0 | R. capsulatus | - | M | R | 60% |  |
| Si | Reactive Ion Etching (RIE) | pillar | 70 | 680 | 180 | 0.10 |  |  | 0 | R. capsulatus | - | M | R | 50% |  |
| Si | Reactive Ion Etching (RIE) | pillar | 100 | 1150 | 280 | 0.09 |  |  | 0 | R. capsulatus | - | M | R | 65% |  |
| Si | Reactive Ion Etching (RIE) | pillar | 170 | 2480 | 400 | 0.07 |  |  | 0 | R. capsulatus | - | M | R | 50% |  |
| Si | Reactive Ion Etching (RIE) | pillar | 220 | 3580 | 380 | 0.06 |  |  | 0 | R. capsulatus | - | M | R | 70% |  |
| Si | Reactive Ion Etching (RIE) | pillar | 320 | 4850 | 490 | 0.07 |  |  | 0 | R. capsulatus | - | M | R | 75% |  |
| Si | Reactive Ion Etching (RIE) | pillar | 490 | 6670 | 600 | 0.07 |  |  | 0 | R. capsulatus | - | M | R | 70% |  |
| Si | Reactive Ion Etching (RIE) | pillar | 50 | 390 | 130 | 0.13 |  |  | 0 | E.coli | - | M | R | 35% |  |
| Si | Reactive Ion Etching (RIE) | pillar | 70 | 680 | 180 | 0.10 |  |  | 0 | E.coli | - | M | R | 65% |  |
| Si | Reactive Ion Etching (RIE) | pillar | 100 | 1150 | 280 | 0.09 |  |  | 0 | E.coli | - | M | R | 45% |  |
| Si | Reactive Ion Etching (RIE) | pillar | 170 | 2480 | 400 | 0.07 |  |  | 0 | E.coli | - | M | R | 50% |  |
| Si | Reactive Ion Etching (RIE) | pillar | 220 | 3580 | 380 | 0.06 |  |  | 0 | E.coli | - | M | R | 75% |  |
| Si | Reactive Ion Etching (RIE) | pillar | 320 | 4850 | 490 | 0.07 |  |  | 0 | E.coli | - | M | R | 70% |  |
| Si | Reactive Ion Etching (RIE) | pillar | 490 | 6670 | 600 | 0.07 |  |  | 0 | E.coli | - | M | R | 81% |  |
| Si H-bD(diamond-coated） | RIE + CVD Diamond coated & Surface terminated | pillar | 300 | 3000 |  | 0.10 |  |  | 55 | E.coli | - | M | R | 33% | .(18) |
| Si O-bD(diamond-coated） | RIE + CVD Diamond coated & Surface terminated | pillar | 300 | 3000 |  | 0.10 |  |  | 5 | E.coli | - | M | R | 20% |  |
| Si F-bD(diamond-coated） | RIE + CVD Diamond coated & Surface terminated | pillar | 300 | 3000 |  | 0.10 |  |  | 137 | E.coli | - | M | R | 50% |  |
| Si NH2-bD(diamond-coated） | RIE + CVD Diamond coated & Surface terminated | pillar | 300 | 3000 |  | 0.10 |  |  | 55 | E.coli | - | M | R | 23% |  |
| Si | Plasma etching | pillar | 21 | 212 | 50 | 0.10 |  |  | 8 | S. aureus | + | N | S | 90% | (19) |
| Si | Plasma etching | pillar | 21 | 212 | 50 | 0.10 |  |  | 8 | P. aeruginosa | - | M | R | 84% |  |
| Si | Plasma etching | pillar | 94 | 475 | 107 | 0.20 |  |  | 8 | S. aureus | + | N | S | 95% |  |
| Si | Plasma etching | pillar | 94 | 475 | 107 | 0.20 |  |  | 8 | P. aeruginosa | - | M | R | 90% |  |
| Si | Plasma etching | pillar | 114 | 610 | 140 | 0.19 |  |  | 8 | S. aureus | + | N | S | 70% |  |
| Si | Plasma etching | pillar | 114 | 610 | 140 | 0.19 |  |  | 8 | P. aeruginosa | - | M | R | 90% |  |
| Si | Plasma etching + PFTE Coated | pillar | 21 | 212 | 50 | 0.10 |  |  | 160 | S. aureus | + | N | S | 97% |  |
| Si | Plasma etching + PFTE Coated | pillar | 21 | 212 | 50 | 0.10 |  |  | 160 | P. aeruginosa | - | M | R | 98% |  |
| Si | Plasma etching + PFTE Coated | pillar | 94 | 475 | 107 | 0.20 |  |  | 160 | S. aureus | + | N | S | 90% |  |
| Si | Plasma etching + PFTE Coated | pillar | 94 | 475 | 107 | 0.20 |  |  | 160 | P. aeruginosa | - | M | R | 98% |  |
| Si | Plasma etching + PFTE Coated | pillar | 114 | 610 | 140 | 0.19 |  |  | 160 | S. aureus | + | N | S | 80% |  |
| Si | Plasma etching + PFTE Coated | pillar | 114 | 610 | 140 | 0.19 |  |  | 160 | P. aeruginosa | - | M | R | 85% |  |
| Si | MacEtch | pillar | 100 | 1100 | 220 | 0.09 |  |  |  | E.coli | - | M | R | 60% | (20) |
| PET | NL + Plasma etching | cone | 54 | 352 | 200 | 0.15 |  |  | 70 | E.coli | - | M | R | 20% | (21) |
| PET | NL + Plasma etching | cone | 54 | 352 | 200 | 0.15 |  |  | 70 | K. pneumoniae | - | N | R | 20% |  |
| PET | NL + Plasma etching | cone | 33 | 388 | 200 | 0.09 |  |  | 70 | E.coli | - | M | R | 24% |  |
| PET | NL + Plasma etching | cone | 33 | 388 | 200 | 0.09 |  |  | 70 | K. pneumoniae | - | N | R | 27% |  |
| PET | NL + Plasma etching | cone | 22 | 498 | 200 | 0.04 |  |  | 70 | E.coli | - | M | R | 30% |  |
| PET | NL + Plasma etching | cone | 22 | 498 | 200 | 0.04 |  |  | 70 | K. pneumoniae | - | N | R | 30% |  |
| PET | NL + Plasma etching | cone | 304 | 419 | 500 | 0.73 |  |  | 75 | E.coli | - | M | R | 10% |  |
| PET | NL + Plasma etching | cone | 304 | 419 | 500 | 0.73 |  |  | 75 | K. pneumoniae | - | N | R | 10% |  |
| PET | NL + Plasma etching | cone | 280 | 456 | 500 | 0.61 |  |  | 75 | E.coli | - | M | R | 12% |  |
| PET | NL + Plasma etching | cone | 280 | 456 | 500 | 0.61 |  |  | 75 | K. pneumoniae | - | N | R | 15% |  |
| PET | NL + Plasma etching | cone | 193 | 529 | 500 | 0.36 |  |  | 75 | E.coli | - | M | R | 14% |  |
| PET | NL + Plasma etching | cone | 193 | 529 | 500 | 0.36 |  |  | 75 | K. pneumoniae | - | N | R | 16% |  |
| CaP | hydrothermal methods | wire | 80 | 1150 | 300 | 0.07 |  |  |  | P. aeruginosa | - | M | R | 70% | (22) |
| CaP | hydrothermal methods | cone | 60 | 640 | 100 | 0.09 |  |  |  | P. aeruginosa | - | M | R | 60% |  |
| Si | deep UV immersion lithography | pillar | 35 | 220 | 90 | 0.16 |  |  |  | P. aeruginosa | - | M | R | 53% | (23) |
| Si | deep UV immersion lithography | pillar | 35 | 220 | 90 | 0.16 |  |  |  | S. aureus | + | N | S | 58% |  |
| Si | deep UV immersion lithography | pillar | 35 | 360 | 90 | 0.10 |  |  |  | P. aeruginosa | - | M | R | 95% |  |
| Si | deep UV immersion lithography | pillar | 35 | 360 | 90 | 0.10 |  |  |  | S. aureus | + | N | S | 83% |  |
| Si | deep UV immersion lithography | pillar | 35 | 420 | 90 | 0.08 |  |  |  | P. aeruginosa | - | M | R | 89% |  |
| Si | deep UV immersion lithography | pillar | 35 | 420 | 90 | 0.08 |  |  |  | S. aureus | + | N | S | 77% |  |
| polycarbonate (PC) | hot-press molding | pillar | 122.9 | 863.7 | 441.5 | 0.14 |  |  |  | P. aeruginosa | - | M | R | 93.80% | (24) |
| polycarbonate (PC) | hot-press molding | pillar | 122.9 | 863.7 | 441.5 | 0.14 |  |  |  | S. aureus | + | N | S | 62.00% |  |
| TiO_2_ | hydrothermal | wire | 25.1 | 1000 |  | 0.03 |  |  |  | P. aeruginosa | - | M | R | 58% | (25) |
| TiO_2_ | hydrothermal | wire | 25.1 | 1000 |  | 0.03 |  |  |  | P. aeruginosa（30h） | - | M | R | 30% |  |
| TiO_2_ -PDMS | double casting | lozenges | 7.28 |  | 5 |  |  |  | 93.65 | E.coli | - | M | R | 67% | (26) |
| TiO_2_-PDMS | double casting | squares | 5.41 |  | 4.82 |  |  |  | 93.65 | E.coli | - | M | R | 80% |  |
| Ti-OH | alkali-heat treatment | pore | 300 |  | 300 |  | 0.69 |  | 20 | S. aureus | + | N | S | 0% | (27) |
| Ti-OH | alkali-heat treatment | pore | 300 |  | 300 |  | 0.69 |  | 20 | P. gingivalis | - | N | R | 0% |  |
| Ti-PAA-NCl | alkali-heat treatment | pore | 200 |  | 200 |  | 0.75 |  | 43.21 | S. aureus | + | N | S | 64% |  |
| Ti-PAA-NCl | alkali-heat treatment | pore | 200 |  | 200 |  | 0.75 |  | 43.21 | P. gingivalis | - | N | R | 42% |  |
| Ti | Low-Temperature Hydrothermal Synthesis | wire-cluster | 23.73 | 289 |  | 0.08 |  |  | 0 | S. aureus | + | N | S | 20.97% | (28) |
| Ti | Low-Temperature Hydrothermal Synthesis | wire-cluster | 23.73 | 289 |  | 0.08 |  |  | 0 | E.coli | - | M | R | 95.79% |  |
| Ti | Low-Temperature Hydrothermal Synthesis | wire/sheet-cluster | 23.01 | 585 |  | 0.04 |  |  | 0 | S. aureus | + | N | S | 69.82% |  |
| Ti | Low-Temperature Hydrothermal Synthesis | wire/sheet-cluster | 23.01 | 585 |  | 0.04 |  |  | 0 | E.coli | - | M | R | 98.60% |  |
| Ti | Low-Temperature Hydrothermal Synthesis | sheet cluster | 69.1 | 1069 |  | 0.06 |  |  | 0 | S. aureus | + | N | S | 3.52% |  |
| Ti | Low-Temperature Hydrothermal Synthesis | sheet cluster | 69.1 | 1069 |  | 0.06 |  |  | 0 | E.coli | - | M | R | 71.67% |  |
| Ti | hydrothermal etching | sheet | 90 | 155 | 158.1 | 0.58 |  |  | 44.1 | P. aeruginosa | - | M | R | 96.60% | (29) |
| Ti | hydrothermal etching | sheet | 90 | 155 | 158.1 | 0.58 |  |  | 44.1 | S. aureus | + | N | S | 24.50% |  |
| Ti | hydrothermal etching | sheet | 92 |  | 185.7 |  |  |  | 40.1 | P. aeruginosa | - | M | R | 98.60% |  |
| Ti | hydrothermal etching | sheet | 92 |  | 185.7 |  |  |  | 40.1 | S. aureus | + | N | S | 36.40% |  |
| Ti | hydrothermal etching | sheet | 90 |  | 213.2 |  |  |  | 34.1 | P. aeruginosa | - | M | R | 98.70% |  |
| Ti | hydrothermal etching | sheet | 90 |  | 213.2 |  |  |  | 34.1 | S. aureus | + | N | S | 21.10% |  |
| Ti | hydrothermal etching | sheet | 115 |  | 229.4 |  |  |  | 20 | P. aeruginosa | - | M | R | 100% |  |
| Ti | hydrothermal etching | sheet | 115 |  | 229.4 |  |  |  | 20 | S. aureus | + | N | S | 65.50% |  |
| Ti | hydrothermal etching | sheet | 120 |  | 250 |  |  |  | 21 | P. aeruginosa | - | M | R | 99.30% |  |
| Ti | hydrothermal etching | sheet | 120 |  | 250 |  |  |  | 21 | S. aureus | + | N | S | 71.40% |  |
| Ti | hydrothermal etching | sheet | 125 |  | 316.2 |  |  |  | 22 | P. aeruginosa | - | M | R | 98.80% |  |
| Ti | hydrothermal etching | sheet | 125 |  | 316.2 |  |  |  | 22 | S. aureus | + | N | S | 87.40% |  |
| Ti | hydrothermal etching | sheet | 124 |  | 301.5 |  |  |  | 15.5 | P. aeruginosa | - | M | R | 99.48% |  |
| Ti | hydrothermal etching | sheet | 124 |  | 301.5 |  |  |  | 15.5 | S. aureus | + | N | S | 71.35 |  |
| Ti | hydrothermal etching | sheet | 180 |  | 333.3 |  |  |  | 13.2 | P. aeruginosa | - | M | R | 40.70% |  |
| Ti | hydrothermal etching | sheet | 180 |  | 333.3 |  |  |  | 13.2 | S. aureus | + | N | S | 2.50% |  |
| Si | metal-assisted chemical etching | cone |  | 1850 | 750 |  |  | 600 | 67.08 | E.coli | - | M | R | 55% | (30) |
| Si | metal-assisted chemical etching | cone |  | 1850 | 750 |  |  | 600 | 67.08 | B. subtilis | + | M | R | 75% |  |
| Si | electron beam induced deposition (EBID) | pillar | 28 | 21 | 40 | 1.33 |  |  |  | E.coli | - | M | R | 0% | (31) |
| Si | electron beam induced deposition (EBID) | pillar | 122 | 183 | 300 | 0.67 |  |  |  | E.coli | - | M | R | 15% |  |
| Si | electron beam induced deposition (EBID) | pillar | 124 | 188 | 299 | 0.66 |  |  |  | E.coli | - | M | R | 8% |  |
| Si | electron beam induced deposition (EBID) | pillar | 126 | 94 | 300 | 1.34 |  |  |  | E.coli | - | M | R | 18% |  |
| ZnO | hydrothermal growth | cone | 47.78 | 1210 |  | 0.04 |  |  |  | P. aeruginosa | - | M | R | 85% | (32) |
| ZnO | hydrothermal growth | cone | 78.9 | 2250 |  | 0.04 |  |  |  | P. aeruginosa | - | M | R | 98% |  |
| ZnO | hydrothermal growth | cone | 125.63 | 3160 |  | 0.04 |  |  |  | P. aeruginosa | - | M | R | 82% |  |
| polyethylene glycol dimethacrylate (PEGDMA) | photopolymerization | pillar | 500 | 500 | 500 | 1.00 |  |  | 52 | E.coli | - | M | R | 99.50% | (33) |
| polyethylene glycol dimethacrylate (PEGDMA) | photopolymerization | pillar | 500 | 500 | 500 | 1.00 |  |  | 52 | B. subtilis | + | M | R | 98.90% |  |
| ethyl acetate-polyDVBAPS | photopolymerization | cone | 100 | 400 | 450 | 0.25 |  |  | 1.9 | P. aeruginosa | - | M | R | 98% | (34) |
| ethyl acetate-polyDVBAPS | photopolymerization | cone | 100 | 400 | 450 | 0.25 |  |  | 1.9 | E.coli | - | M | R | 98% |  |
| ethyl acetate-polyDVBAPS | photopolymerization | cone | 100 | 400 | 450 | 0.25 |  |  | 15.8 | P. aeruginosa | - | M | R | 98% |  |
| ethyl acetate-polyDVBAPS | photopolymerization | cone | 100 | 400 | 450 | 0.25 |  |  | 15.8 | E.coli | - | M | R | 98% |  |
| ethyl acetate-polyDVBAPS | photopolymerization | cone | 100 | 400 | 450 | 0.25 |  |  | 4.5 | P. aeruginosa | - | M | R | 98% |  |
| ethyl acetate-polyDVBAPS | photopolymerization | cone | 100 | 400 | 450 | 0.25 |  |  | 4.5 | E.coli | - | M | R | 98% |  |
| Ti | electrochemical characterization | tube | 70 | 550 | 70 | 0.13 |  |  | 52 | E.coli | - | M | R | 5% | (35) |
| Ti | electrochemical characterization | tube | 70 | 550 | 70 | 0.13 |  |  | 52 | S. aureus | + | N | S | 10% |  |
| Ti | electrochemical characterization | tube | 70 | 550 | 70 | 0.13 |  |  | 52 | E.faecalis | + | N | S | 5% |  |
| Ti | electrochemical characterization | tube | 70 | 550 | 70 | 0.13 |  |  | 52 | P. aeruginosa | - | M | R | 4% |  |
| Ti | electrochemical characterization | tube | 70 | 550 | 70 | 0.13 |  |  | 52 | B. subtilis | + | M | R | 4% |  |
| Ti | electrochemical characterization | tube | 70 | 550 | 70 | 0.13 |  |  | 14 | E.coli | - | M | R | 55% |  |
| Ti | electrochemical characterization | tube | 70 | 550 | 70 | 0.13 |  |  | 14 | S. aureus | + | N | S | 58% |  |
| Ti | electrochemical characterization | tube | 70 | 550 | 70 | 0.13 |  |  | 14 | E.faecalis | + | N | S | 50% |  |
| Ti | electrochemical characterization | tube | 70 | 550 | 70 | 0.13 |  |  | 14 | P. aeruginosa | - | M | R | 25% |  |
| Ti | electrochemical characterization | tube | 70 | 550 | 70 | 0.13 |  |  | 14 | B. subtilis | + | M | R | 58% |  |
| Ti | electrochemical characterization | tube | 70 | 550 | 70 | 0.13 |  |  | 42 | E.coli | - | M | R | 62% |  |
| Ti | electrochemical characterization | tube | 70 | 550 | 70 | 0.13 |  |  | 42 | S. aureus | + | N | S | 64% |  |
| Ti | electrochemical characterization | tube | 70 | 550 | 70 | 0.13 |  |  | 42 | E.faecalis | + | N | S | 60% |  |
| Ti | electrochemical characterization | tube | 70 | 550 | 70 | 0.13 |  |  | 42 | P. aeruginosa | - | M | R | 40% |  |
| Ti | electrochemical characterization | tube | 70 | 550 | 70 | 0.13 |  |  | 42 | B. subtilis | + | M | R | 65% |  |
| Ti | hydrothermal treatment | cone |  | 300 |  |  |  |  |  | P. aeruginosa | - | M | R | 74% | (36) |
| Ti | hydrothermal treatment | cone |  | 300 |  |  |  |  |  | S. aureus | + | N | S | 12% |  |
| Ti | hydrothermal treatment | cone |  | 1125 |  |  |  |  |  | P. aeruginosa | - | M | R | 76% |  |
| Ti | hydrothermal treatment | cone |  | 1125 |  |  |  |  |  | S. aureus | + | N | S | 51% |  |
| Pd | hydrothermal method | cube | 10 | 10 |  | 1.00 |  |  |  | S. aureus | + | N | S | 100% | (37) |
| Pd | hydrothermal method | cube | 10 | 10 |  | 1.00 |  |  |  | E.coli | - | M | R | 95% |  |
| Pd | hydrothermal method | octahedral | 10 | 10 |  | 1.00 |  |  |  | S. aureus | + | N | S | 80% |  |
| Pd | hydrothermal method | octahedral | 10 | 10 |  | 1.00 |  |  |  | E.coli | - | M | R | 98% |  |
| PS-b-P2VP | self-assembly | pillar | 66.05 | 27.5 |  | 2.40 | 3 |  |  | E.coli | - | M | R | 80% | (38) |
| PS-b-P2VP | self-assembly | pillar | 66.05 | 27.5 |  | 2.40 | 3 |  |  | S. aureus | + | N | S | 5% |  |
| PS-b-P2VP | self-assembly | pillar | 88.45 | 40.3 |  | 2.19 | 4 |  |  | E.coli | - | M | R | 95% |  |
| PS-b-P2VP | self-assembly | pillar | 88.45 | 40.3 |  | 2.19 | 4 |  |  | S. aureus | + | N | S | 20% |  |
| Si | ultraviolet nanoimprint lithography | pillar | 55 | 163 | 170 | 3.00 |  |  | 73.7 | E.coli | - | M | R | 64% | (39) |
| Si | ultraviolet nanoimprint lithography | pillar | 77 | 205 | 200 | 2.70 |  |  | 73.7 | E.coli | - | M | R | 79% |  |
| Si | ultraviolet nanoimprint lithography | pillar | 174 | 272 | 400 | 1.60 |  |  | 73.7 | E.coli | - | M | R | 8% |  |
| Si | ultraviolet nanoimprint lithography | pillar | 178 | 285 | 500 | 1.60 |  |  | 73.7 | E.coli | - | M | R | 5% |  |
| Si | ultraviolet nanoimprint lithography | pillar | 41 | 145 | 170 | 3.50 |  |  | 83.7 | E.coli | - | M | R | 82% |  |
| Si | ultraviolet nanoimprint lithography | pillar | 65 | 195 | 200 | 3.00 |  |  | 83.7 | E.coli | - | M | R | 50.80% |  |
| Si | ultraviolet nanoimprint lithography | pillar | 168 | 247 | 400 | 1.50 |  |  | 83.7 | E.coli | - | M | R | 18% |  |
| Si | ultraviolet nanoimprint lithography | pillar | 171 | 243 | 500 | 1.40 |  |  | 83.7 | E.coli | - | M | R | 5% |  |
| Si | ultraviolet nanoimprint lithography | pillar | 72 | 166 | 170 | 2.30 |  |  | 74 | E.coli | - | M | R | 20% |  |
| Si | ultraviolet nanoimprint lithography | pillar | 82 | 211 | 200 | 2.60 |  |  | 74 | E.coli | - | M | R | 7% |  |
| Si | ultraviolet nanoimprint lithography | pillar | 181 | 256 | 400 | 1.40 |  |  | 74 | E.coli | - | M | R | 12% |  |
| Si | ultraviolet nanoimprint lithography | pillar | 185 | 277 | 500 | 1.50 |  |  | 74 | E.coli | - | M | R | 6% |  |
| Si | ultraviolet nanoimprint lithography | pillar | 67 | 192 | 170 | 2.80 |  |  | 74 | E.coli | - | M | R | 8% |  |
| Si | ultraviolet nanoimprint lithography | pillar | 90 | 210 | 200 | 2.30 |  |  | 74 | E.coli | - | M | R | 5% |  |
| Si | ultraviolet nanoimprint lithography | pillar | 185 | 260 | 400 | 1.40 |  |  | 74 | E.coli | - | M | R | 11% |  |
| Si | ultraviolet nanoimprint lithography | pillar | 186 | 264 | 500 | 1.40 |  |  | 74 | E.coli | - | M | R | 10% |  |
| PS-block-PMMA | Atomic Layer Deposition (ALD) | pillar | 44 | 11 | 66 | 4.00 | 2.8 |  | 51 | E.coli | - | M | R | 95% | (40) |
| TiO_2_ | Atomic Layer Deposition (ALD) | pillar | 46 | 14 | 66 | 3.29 | 5.5 |  | 52 | E.coli | - | M | R | 95% |  |
| PS-block-PMMA | Atomic Layer Deposition (ALD) | pillar | 44 | 11 | 66 | 4.00 | 2.8 |  | 51 | L. monocytogenes | + | M | R | 5.80% |  |
| TiO_2_ | Atomic Layer Deposition (ALD) | pillar | 46 | 14 | 66 | 3.29 | 5.5 |  | 52 | L. monocytogenes | + | M | R | 69.90% |  |
| ZnO | Hydrothermal growth | cone | 118 | 200 |  | 0.59 |  |  |  | E.coli | - | M | R | 100% | (41) |
| ZnO | Hydrothermal growth | cone | 118 | 200 |  | 0.59 |  |  |  | S. aureus | + | N | S | 100% |  |
| ZnO | Hydrothermal growth | cone | 75.3 | 200 |  | 0.38 |  |  |  | E.coli | - | M | R | 100% |  |
| ZnO | Hydrothermal growth | cone | 75.3 | 200 |  | 0.38 |  |  |  | S. aureus | + | N | S | 100% |  |
| ZnO | Hydrothermal growth | pillar | 28.5 | 200 |  | 0.14 |  |  |  | E.coli | - | M | R | 100% |  |
| ZnO | Hydrothermal growth | pillar | 28.5 | 200 |  | 0.14 |  |  |  | S. aureus | + | N | S | 100% |  |
| Si | ion etching | pillar | 50.00 | 390 | 130 | 0.13 |  |  | 10 | E.coli | - | M | R | 33% | (42) |
| Si | ion etching | pillar | 50.00 | 390 | 130 | 0.13 |  |  | 10 | R.capsulatus | - | M | S | 75% |  |
| Si | ion etching | cone | 220.00 | 3600 | 380 | 0.06 |  |  | 10 | E.coli | - | M | R | 79% |  |
| Si | ion etching | cone | 220.00 | 3600 | 380 | 0.06 |  |  | 10 | R.capsulatus | - | M | S | 82% |  |
| Si | ion etching | pillar | 35 | 260 | 90 | 0.13 |  |  |  | S. aureus | + | N | S | 80% | (43) |
| Si | ion etching | pillar | 35 | 260 | 90 | 0.13 |  |  |  | P. aeruginosa | - | M | R | 89% |  |
| Si | ion etching | pillar | 90 | 260 | 90 | 0.35 |  |  |  | S. aureus | + | N | S | 97% |  |
| Si | ion etching | pillar | 90 | 260 | 90 | 0.35 |  |  |  | P. aeruginosa | - | M | R | 98% |  |
| Ti | ion etching | spike | 30 | 112 | 185 | 0.27 |  |  |  | E.coli | - | M | R | 68% | (44) |
| Ti | ion etching | spike | 30 | 112 | 185 | 0.27 |  |  |  | P. aeruginosa | - | M | R | 98% |  |
| Ti | ion etching | spike | 30 | 112 | 185 | 0.27 |  |  |  | S. aureus | + | N | S | 30% |  |
| Ti | ion etching | spike | 94 | 185 | 190 | 0.51 |  |  |  | E.coli | - | M | R | 76% |  |
| Ti | ion etching | spike | 94 | 185 | 190 | 0.51 |  |  |  | P. aeruginosa | - | M | R | 95% |  |
| Ti | ion etching | spike | 94 | 185 | 190 | 0.51 |  |  |  | S. aureus | + | N | S | 10% |  |
| Ti | ion etching | spike | 62 | 367 | 182 | 0.17 | 71.4 |  | 7.6 | E.coli | - | M | R | 80% |  |
| Ti | ion etching | spike | 62 | 367 | 182 | 0.17 | 71.4 |  | 7.6 | P. aeruginosa | - | M | R | 98% |  |
| Ti | ion etching | spike | 62 | 367 | 182 | 0.17 | 71.4 |  | 7.6 | S. aureus | + | N | S | 55% |  |
| Ti | ion etching | spike | 83 | 425 | 224 | 0.20 |  |  |  | E.coli | - | M | R | 70% |  |
| Ti | ion etching | spike | 83 | 425 | 224 | 0.20 |  |  |  | P. aeruginosa | - | M | R | 94% |  |
| Ti | ion etching | spike | 83 | 425 | 224 | 0.20 |  |  |  | S. aureus | + | N | S | 55% |  |
| Ti | ion etching | spike | 65 | 122 | 330 | 0.53 |  |  |  | E.coli | - | M | R | 60% |  |
| Ti | ion etching | spike | 65 | 122 | 330 | 0.53 |  |  |  | P. aeruginosa | - | M | R | 87% |  |
| Ti | ion etching | spike | 65 | 122 | 330 | 0.53 |  |  |  | S. aureus | + | N | S | 58% |  |
| Ti | ion etching | spike | 71 | 242 | 453 | 0.29 |  |  |  | E.coli | - | M | R | 50% |  |
| Ti | ion etching | spike | 71 | 242 | 453 | 0.29 |  |  |  | P. aeruginosa | - | M | R | 84% |  |
| Ti | ion etching | spike | 71 | 242 | 453 | 0.29 |  |  |  | S. aureus | + | N | S | 28% |  |
| Ti | ion etching | spike | 85 | 207 | 500 | 0.41 |  |  |  | E.coli | - | M | R | 40% |  |
| Ti | ion etching | spike | 85 | 207 | 500 | 0.41 |  |  |  | P. aeruginosa | - | M | R | 95% |  |
| Ti | ion etching | spike | 85 | 207 | 500 | 0.41 |  |  |  | S. aureus | + | N | S | 32% |  |
| Ti | ion etching | spike | 83 | 340 | 544 | 0.24 | 61.5 |  | 9.7 | E.coli | - | M | R | 62% |  |
| Ti | ion etching | spike | 83 | 340 | 544 | 0.24 | 61.5 |  | 9.7 | P. aeruginosa | - | M | R | 96% |  |
| Ti | ion etching | spike | 83 | 340 | 544 | 0.24 | 61.5 |  | 9.7 | S. aureus | + | N | S | 64% |  |
| Carbon | chemical vapor deposition | tube | 10 | 32000 | 75 | 0.0003 |  |  | 148.6 | P. aeruginosa | - | M | R | 100% | (45) |
| Carbon | chemical vapor deposition | tube | 10 | 32000 | 75 | 0.0003 |  |  | 148.6 | S. aureus | + | N | S | 18% |  |
| Carbon | chemical vapor deposition | tube | 10 | 32000 | 75 | 0.0003 |  |  | 147.93 | P. aeruginosa | - | M | R | 90% |  |
| Carbon | chemical vapor deposition | tube | 10 | 32000 | 75 | 0.0003 |  |  | 147.93 | S. aureus | + | N | S | 17% |  |
| Carbon | chemical vapor deposition | tube | 10 | 32000 | 75 | 0.0003 |  |  | 45.5 | P. aeruginosa | - | M | R | 92% |  |
| Carbon | chemical vapor deposition | tube | 10 | 32000 | 75 | 0.0003 |  |  | 45.5 | S. aureus | + | N | S | 10% |  |
| Carbon | chemical vapor deposition | tube | 10 | 1000 | 75 | 0.0100 |  |  | 149.5 | P. aeruginosa | - | M | R | 75% |  |
| Carbon | chemical vapor deposition | tube | 10 | 1000 | 75 | 0.0100 |  |  | 149.5 | S. aureus | + | N | S | 68% |  |
| Carbon | chemical vapor deposition | tube | 10 | 1000 | 75 | 0.0100 |  |  | 137.1 | P. aeruginosa | - | M | R | 90% |  |
| Carbon | chemical vapor deposition | tube | 10 | 1000 | 75 | 0.0100 |  |  | 137.1 | S. aureus | + | N | S | 85% |  |
| Carbon | chemical vapor deposition | tube | 10 | 1000 | 75 | 0.0100 |  |  | 21.4 | P. aeruginosa | - | M | R | 100% |  |
| Carbon | chemical vapor deposition | tube | 10 | 1000 | 75 | 0.0100 |  |  | 21.4 | S. aureus | + | N | S | 50% |  |
|  |  |  |  |  |  |  |  |  |  |  |  |  |  |  |  |

References：

1. Linklater DP, Juodkazis S, Crawford RJ, Ivanova EP. Mechanical inactivation of Staphylococcus aureus and Pseudomonas aeruginosa by titanium substrata with hierarchical surface structures. Materialia. 2019 Mar 1;5:100197.

2. Hasan J, Jain S, Chatterjee K. Nanoscale Topography on Black Titanium Imparts Multi-biofunctional Properties for Orthopedic Applications. Sci Rep. 2017 Jan 23;7(1):41118.

3. Jaggessar A, Mathew A, Wang H, Tesfamichael T, Yan C, Yarlagadda PK. Mechanical, bactericidal and osteogenic behaviours of hydrothermally synthesised TiO2 nanowire arrays. J Mech Behav Biomed Mater. 2018 Apr 1;80:311–9.

4. Diu T, Faruqui N, Sjöström T, Lamarre B, Jenkinson HF, Su B, et al. Cicada-inspired cell-instructive nanopatterned arrays. Sci Rep. 2014 Nov 20;4(1):7122.

5. Jaggessar A, Mathew A, Tesfamichael T, Wang H, Yan C, Yarlagadda PK. Bacteria Death and Osteoblast Metabolic Activity Correlated to Hydrothermally Synthesised TiO2 Surface Properties. Molecules. 2019 Jan;24(7):1201.

6. Bhadra CM, Khanh Truong V, Pham VTH, Al Kobaisi M, Seniutinas G, Wang JY, et al. Antibacterial titanium nano-patterned arrays inspired by dragonfly wings. Sci Rep. 2015 Nov 18;5(1):16817.

7. Sengstock C, Lopian M, Motemani Y, Borgmann A, Khare C, Buenconsejo PJS, et al. Structure-related antibacterial activity of a titanium nanostructured surface fabricated by glancing angle sputter deposition. Nanotechnology. 2014 Apr;25(19):195101.

8. Izquierdo-Barba I, García-Martín JM, Álvarez R, Palmero A, Esteban J, Pérez-Jorge C, et al. Nanocolumnar coatings with selective behavior towards osteoblast and Staphylococcus aureus proliferation. Acta Biomater. 2015 Mar 15;15:20–8.

9. Hizal F, Zhuk I, Sukhishvili S, Busscher HJ, van der Mei HC, Choi CH. Impact of 3D Hierarchical Nanostructures on the Antibacterial Efficacy of a Bacteria-Triggered Self-Defensive Antibiotic Coating. ACS Appl Mater Interfaces. 2015 Sep 16;7(36):20304–13.

10. Ercan B, Taylor E, Alpaslan E, Webster TJ. Diameter of titanium nanotubes influences anti-bacterial efficacy. Nanotechnology. 2011 Jun;22(29):295102.

11. Bright R, Hayles A, Fernandes D, Visalakshan RM, Ninan N, Palms D, et al. In Vitro Bactericidal Efficacy of Nanostructured Ti6Al4V Surfaces is Bacterial Load Dependent. ACS Appl Mater Interfaces. 2021 Aug 18;13(32):38007–17.

12. Li X, Qi M, Sun X, Weir MD, Tay FR, Oates TW, et al. Surface treatments on titanium implants via nanostructured ceria for antibacterial and anti-inflammatory capabilities. Acta Biomater. 2019 Aug 1;94:627–43.

13. Huang LZY, Elbourne A, Shaw ZL, Cheeseman S, Goff A, Orrell-Trigg R, et al. Dual-action silver functionalized nanostructured titanium against drug resistant bacterial and fungal species. J Colloid Interface Sci. 2022 Dec 15;628:1049–60.

14. Liu P, Zhao Z, Tang J, Wang A, Zhao D, Yang Y. Early Antimicrobial Evaluation of Nanostructured Surfaces Based on Bacterial Biological Properties. ACS Biomater Sci Eng. 2022 Nov 14;8(11):4976–86.

15. Linklater DP, Nguyen HKD, Bhadra CM, Juodkazis S, Ivanova EP. Influence of nanoscale topology on bactericidal efficiency of black silicon surfaces. Nanotechnology. 2017 Jun 16;28(24):245301.

16. Ivanova EP, Hasan J, Webb HK, Gervinskas G, Juodkazis S, Truong VK, et al. Bactericidal activity of black silicon. Nat Commun. 2013 Nov 26;4(1):2838.

17. Michalska M, Gambacorta F, Divan R, Aranson IS, Sokolov A, Noirot P, et al. Tuning antimicrobial properties of biomimetic nanopatterned surfaces. Nanoscale. 2018 Apr 5;10(14):6639–50.

18. Dunseath O, Smith EJW, Al-Jeda T, Smith JA, King S, May PW, et al. Studies of Black Diamond as an antibacterial surface for Gram Negative bacteria: the interplay between chemical and mechanical bactericidal activity. Sci Rep. 2019 Jun 19;9(1):8815.

19. Linklater DP, Juodkazis S, Rubanov S, Ivanova EP. Comment on “Bactericidal Effects of Natural Nanotopography of Dragonfly Wing on Escherichia coli”. ACS Appl Mater Interfaces. 2017 Sep 6;9(35):29387–93.

20. Hu H, Siu VS, Gifford SM, Kim S, Lu M, Meyer P, et al. Bio-inspired silicon nanospikes fabricated by metal-assisted chemical etching for antibacterial surfaces. Appl Phys Lett. 2017 Dec 21;111(25):253701.

21. Hazell G, Fisher LE, Murray WA, Nobbs AH, Su B. Bioinspired bactericidal surfaces with polymer nanocone arrays. J Colloid Interface Sci. 2018 Oct 15;528:389–99.

22. Iglesias-Fernandez M, Buxadera-Palomero J, Sadowska JM, Espanol M, Ginebra MP. Implementation of bactericidal topographies on biomimetic calcium phosphates and the potential effect of its reactivity. Biomater Adv. 2022 May 1;136:212797.

23. Ivanova EP, Linklater DP, Werner M, Baulin VA, Xu X, Vrancken N, et al. The multi-faceted mechano-bactericidal mechanism of nanostructured surfaces. Proc Natl Acad Sci. 2020 Jun 9;117(23):12598–605.

24. Liu Z, Yi Y, Wang S, Dou H, Fan Y, Tian L, et al. Bio-Inspired Self-Adaptive Nanocomposite Array: From Non-antibiotic Antibacterial Actions to Cell Proliferation. ACS Nano. 2022 Oct 25;16(10):16549–62.

25. Tsimbouri PM, Fisher L, Holloway N, Sjostrom T, Nobbs AH, Meek RMD, et al. Osteogenic and bactericidal surfaces from hydrothermal titania nanowires on titanium substrates. Sci Rep. 2016 Nov 18;6(1):36857.

26. Vieira A, Rodríguez-Lorenzo L, Leonor IB, Reis RL, Espiña B, dos Santos MB. Innovative Antibacterial, Photocatalytic, Titanium Dioxide Microstructured Surfaces Based on Bacterial Adhesion Enhancement. ACS Appl Bio Mater. 2023 Feb 20;6(2):754–64.

27. Wu S, Xu J, Zou L, Luo S, Yao R, Zheng B, et al. Long-lasting renewable antibacterial porous polymeric coatings enable titanium biomaterials to prevent and treat peri-implant infection. Nat Commun. 2021 Jun 3;12(1):3303.

28. Zhao L, Liu T, Li X, Cui Q, Wu Q, Wang X, et al. Low-Temperature Hydrothermal Synthesis of Novel 3D Hybrid Nanostructures on Titanium Surface with Mechano-bactericidal Performance. ACS Biomater Sci Eng. 2021 Jun 14;7(6):2268–78.

29. Wandiyanto JV, Tamanna T, Linklater DP, Truong VK, Al Kobaisi M, Baulin VA, et al. Tunable morphological changes of asymmetric titanium nanosheets with bactericidal properties. J Colloid Interface Sci. 2020 Feb 15;560:572–80.

30. Saini SK, Halder M, Singh Y, Nair RV. Bactericidal Characteristics of Bioinspired Nontoxic and Chemically Stable Disordered Silicon Nanopyramids. ACS Biomater Sci Eng. 2020 May 11;6(5):2778–86.

31. Widyaratih DS, Hagedoorn PL, Otten LG, Ganjian M, Tümer N, Apachitei I, et al. Towards osteogenic and bactericidal nanopatterns? Nanotechnology. 2019 Mar;30(20):20LT01.

32. Yi Y, Jiang R, Liu Z, Dou H, Song L, Tian L, et al. Bioinspired nanopillar surface for switchable mechano-bactericidal and releasing actions. J Hazard Mater. 2022 Jun 15;432:128685.

33. Kim HK, Cho YS, Park HH. PEGDMA-Based Pillar-Shape Nanostructured Antibacterial Films Having Mechanical Robustness. ACS Appl Bio Mater. 2022 Jun 20;5(6):3006–12.

34. Liu Z, Yi Y, Song L, Chen Y, Tian L, Zhao J, et al. Biocompatible mechano-bactericidal nanopatterned surfaces with salt-responsive bacterial release. Acta Biomater. 2022 Mar 15;141:198–208.

35. Ungureanu C, Dumitriu C, Popescu S, Enculescu M, Tofan V, Popescu M, et al. Enhancing antimicrobial activity of TiO2/Ti by torularhodin bioinspired surface modification. Bioelectrochemistry. 2016 Feb 1;107:14–24.

36. Elliott DT, Wiggins RJ, Dua R. Bioinspired antibacterial surface for orthopedic and dental implants. J Biomed Mater Res B Appl Biomater. 2021;109(7):973–81.

37. Fang G, Li W, Shen X, Perez-Aguilar JM, Chong Y, Gao X, et al. Differential Pd-nanocrystal facets demonstrate distinct antibacterial activity against Gram-positive and Gram-negative bacteria. Nat Commun. 2018 Jan 9;9(1):129.

38. Fontelo R, Soares da Costa D, Reis RL, Novoa-Carballal R, Pashkuleva I. Bactericidal nanopatterns generated by block copolymer self-assembly. Acta Biomater. 2020 Aug 1;112:174–81.

39. Lohmann SC, Tripathy A, Milionis A, Keller A, Poulikakos D. Effect of Flexibility and Size of Nanofabricated Topographies on the Mechanobactericidal Efficacy of Polymeric Surfaces. ACS Appl Bio Mater. 2022 Apr 18;5(4):1564–75.

40. Salatto D, Huang Z, Benziger PT, Carrillo JMY, Bajaj Y, Gauer A, et al. Structure-Based Design of Dual Bactericidal and Bacteria-Releasing Nanosurfaces. ACS Appl Mater Interfaces. 2023 Jan 18;15(2):3420–32.

41. Xie Y, Qu X, Li J, Li D, Wei W, Hui D, et al. Ultrafast physical bacterial inactivation and photocatalytic self-cleaning of ZnO nanoarrays for rapid and sustainable bactericidal applications. Sci Total Environ. 2020 Oct 10;738:139714.

42. Michalska M, Divan R, Noirot P, Laible PD. Antimicrobial properties of nanostructured surfaces – demonstrating the need for a standard testing methodology. Nanoscale. 2021 Oct 28;13(41):17603–14.

43. Zahir T, Pesek J, Franke S, Van Pee J, Rathore A, Smeets B, et al. Model-Driven Controlled Alteration of Nanopillar Cap Architecture Reveals its Effects on Bactericidal Activity. Microorganisms. 2020 Jan 28;8(2):186.

44. Bright R, Hayles A, Wood J, Ninan N, Palms D, Visalakshan RM, et al. Bio-Inspired Nanostructured Ti-6Al-4V Alloy: The Role of Two Alkaline Etchants and the Hydrothermal Processing Duration on Antibacterial Activity. Nanomaterials. 2022 Jan;12(7):1140.

45. Linklater DP, De Volder M, Baulin VA, Werner M, Jessl S, Golozar M, et al. High Aspect Ratio Nanostructures Kill Bacteria via Storage and Release of Mechanical Energy. ACS Nano. 2018 Jul 24;12(7):6657–67.
